# Several orphan solute carriers functionally identified as organic cation transporters: Substrates specificity compared with known cation transporters

**DOI:** 10.1016/j.jbc.2024.107629

**Published:** 2024-08-03

**Authors:** Kyra-Elisa Maria Redeker, Jürgen Brockmöller

**Affiliations:** Institute of Clinical Pharmacology, University Medical Center Göttingen, Georg-August-University Göttingen, Göttingen, Germany

**Keywords:** solute carrier, orphan transporters, organic cation transporter, proton-coupled organic cation antiporter, substrate specificity, SLC35G3, SLC35G4, SLC35F5, SLC38A10

## Abstract

Organic cations comprise a significant part of medically relevant drugs and endogenous substances. Such substances need organic cation transporters for efficient transfer *via* cell membranes. However, the membrane transporters of most natural or synthetic organic cations are still unknown. To identify these transporters, genes of 10 known OCTs and 18 orphan solute carriers (SLC) were overexpressed in HEK293 cells and characterized concerning their transport activities with a broad spectrum of low molecular weight substances emphasizing organic cations. Several SLC35 transporters and SLC38A10 significantly enhanced the transport of numerous relatively hydrophobic organic cations. Significant organic cation transport activities have been found in gene families classified as transporters of other substance classes. For instance, SLC35G3 and SLC38A10 significantly accelerated the uptake of several cations, such as clonidine, 3,4-methylenedioxymethamphetamine, and nicotine, which are known as substrates of a thus far genetically unidentified proton/organic cation antiporter. The transporters SLC35G4 and SLC35F5 stood out by their significantly increased choline uptake, and several other SLC transported choline together with a broader spectrum of organic cations. Overall, there are many more polyspecific organic cation transporters than previously estimated. Several transporters had one predominant substrate but accepted some other cationic substrates, and others showed no particular preference for one substrate but transported several organic cations. The role of these transporters in biology and drug therapy remains to be elucidated.

Transport proteins facilitate the exchange of endogenous and exogenous substances across biological membranes. Most transporters mediating membrane transport of small organic molecules can be divided into two groups: the ATP-binding cassette drug transporter on the one side and the solute carrier (SLC), facilitating passive transport without energy consumption on the other side. The family of SLCs consists of 446 members divided into 70 families (1). About 30% of these SLCs are so-called orphan transporters for which the substrates and the biological roles are still unknown (1).

Here, we focus on carriers accelerating the membrane transport of organic cations. Organic cations are partially or entirely positively charged at the pH in the extracellular (interstitial) fluids of humans and many other living organisms. Because of their charge, they diffuse poorly *via* biological membranes. Better knowledge concerning which substance is transported by which transport protein may contribute to a better understanding of cell functions and the beneficial or adverse effects of synthetic organic compounds like drugs.

Several proteins accelerate the transmembrane transport of organic cations. Since the discovery of the organic cation transporter one (OCT1) (2), several other so-called polyspecific organic cation transporters have been described, including OCT2 and OCT3, but also the multidrug and toxin extrusion transporters MATE1 and MATE2K (Table 1). They are all polyspecific, indicating that each of these transporters has a broad but distinct and not yet completely understood substrate specificity. Recent advances in structural biology have significantly increased our understanding of substrate binding and membrane translocation mechanisms (3, 4). However, although the transporters for several hundred organic cations are well characterized (5, 6, 7, 8, 9, 10, 11, 12), for the vast majority of endogenous and exogenous organic cations, the relevant membrane transporters are not yet known. Based on several criteria, carrier-mediated transport of cationic drugs through the endothelial cells at the blood–brain barrier is well established. Numerous studies have shown that the transport of many organic cations in endothelial cells of the blood–brain barrier is mediated by a proton-organic cation antiporter (H^+^/OC antiporter) (13, 14). However, the proteins underlying that H^+^/OC antiport activity are still unknown, and the present investigation aims to identify them.

**Table 1:** Summary of investigated SLCs

Name	Other names	Chromosome location	Amino acids	Transmembrane helices	Tissue expression
SLC22A1	OCT1	6q25.3	554	12	Liver
SLC22A2	OCT2	6q25.3	555	12	Kidneys
SLC22A3	OCT3	6q25.3	556	12	Broad, including brain, heart, liver, kidneys
SLC22A15	FLIPT1	1p13.1	547	12	Bone marrow, brain
SLC22A16	OCT6; OAT6; FLIPT2	6q21-q22.2	577	12	Bone marrow, testes
SLC22A4	OCTN1	5q31.1	551	12	Bone marrow, kidneys, small intestine, brain
SLC22A5	OCTN2	5q31.1	557	12	Muscle tissue, kidneys, brain
SLC22A17	BOCT	14q11.2	649	11	Brain
SLC22A18	ORCTL2	11p15.4	424	10	Gastrointestinal tract, liver, kidneys
SLC38A10	PP1744	17q25.3	1119	10	Broad, including pancreas, liver, brain, and others
SLC35G4	AMAC1L1	18p11.21	338	10	Broad, including pancreas, muscle, brain
SLC35G5	AMAC1L2	8p23.1	338	10	Testes, brain (minor)
SLC35G6	AMAC1L3, TMEM21B	17p13.1	338	10	Testes, brain (minor)
SLC35G3	AMAC1, TMEM21A	17q12	338	10	Testes
SLC35G2	TMEM22	3q22.3	412	10	Broad, including brain, endocrine tissue
SLC35E2A	SLC35E2	1p36.33	266	4	Fetus brain
SLC35E1		19p13.11	410	9	Broad, including brain, pancreas, muscle
SLC35E4		22q12.2	350	10	Testes, muscle, brain (minor)
SLC47A1	MATE1	17p11.2	570	13	Broad, including endocrine tissues, kidneys, liver, brain (minor)
SLC47A2	MATE2-K	17p11.2	602	13	Kidney, brain (minor)
SLC35F3		1q42.2	421	10	Brain
SLC35F4	C14orf36	14q22.3-q23.1	521	10	Brain, retina
SLC35F5	NS5ATP3	2q14.1	523	10	Broad, including brain, endocrine tissues, kidneys, liver, skeletal muscle
SLC35F2	HSNOV1	11q22.3	374	10	Broad, including gastrointestinal tract, endocrine tissue, brain (minor)
SLC19A2	THTR1	1q24.2	497	12	Broad, including muscle tissue, brain (minor)
SLC19A3	THTR2	2q36.3	497	12	Broad, including connective tissues, brain (minor)
SLC44A1	CTL1	9q31.1-q31.2	657	9	Broad, including brain
SLC44A2	CTL2	19p13.2	706	10	Broad, including placenta, muscle tissue, and brain

SLC47A2: for this study, the kidney-specific isoform was used which leads to the protein also called MATE2K; References accessed on May 2nd 2024: https://www.uniprot.org (75); https://www.ncbi.nlm.nih.gov (52); https://www.proteinatlas.org (50); Nishimura *et al.* 2009 (51).

These unknown organic cation transporters may be primarily polyspecific SLCs, but also specific SLCs have to be considered, which are known to be, and named specifically, for their one substrate. These transporters include the high-affinity monoamine transporters (SLC6A2/3/4), the thiamine transporters (SLC19A2/3), and the high-affinity choline transporter (SLC5A7) (15, 16, 17, 18). Genetic deficiency of such specific transporters is often associated with severe disease symptoms (19, 20). Many of these transporters, known as carriers of specific substrates, may additionally transport numerous other substrates chemically similar to the name-giving substrates (21, 22).

Here, we investigated comparatively 28 SLCs as potential transporters for organic cations. Eighteen of these transporters can be considered orphan transporters. We wanted to provide a better understanding of their function and substrate spectrum. For comparison, transport of the same substrates was also measured with known transporters. Since we could not exclude that some of these transporters were organic anion transporters, we also applied a few anionic substrates known to be transported by several organic anion transporters. The selection of the genes was based on several criteria. First, we considered expression in blood–brain barrier epithelial cells because we were particularly interested in identifying gene(s) coding for the so-called proton organic-cation antiporter (14). However, since even low expression does not exclude relevant activity depending on the kinetic constants, we also included genes with high homology to known organic cation transporters. We excluded all those well characterized as transporters of inorganic ions, amino acids, nucleotides, and organic cations. All 28 SLCs were stably expressed in HEK293 cells, and we measured influx transport with a broad spectrum of structurally diverse positively and negatively charged substances, amino acids, and numerous drugs that are substrates of the H^+^/OC antiporter.

## Results

The coding sequence of 10 known transporters of organic cations and 18 orphan (or thus far poorly functionally characterized) SLCs were integrated into a pcDNA5/FRT vector *via* restriction enzyme digestion cloning. After transformation into *Escherichia coli* TOP10, the correct coding sequence was verified by Sanger sequencing. Their correct genomic integration in HEK293 cells was validated with a short-range PCR of the vector backbone and by amplifying the entire gene-of-interest within the HEK293 genome *via* a long-range PCR, as described in the methods section. Sequence alignment of 26 of the 28 SLC genes is illustrated in Fig. S1. Sequences of SLC35E2A and SLC38A10 are not shown in this alignment because of their unique genetic architectures (four transmembrane helices and huge interhelical loops, respectively).

### Comparative transport activities of orphan transporters and known organic cation transporters

**Figure 1:**
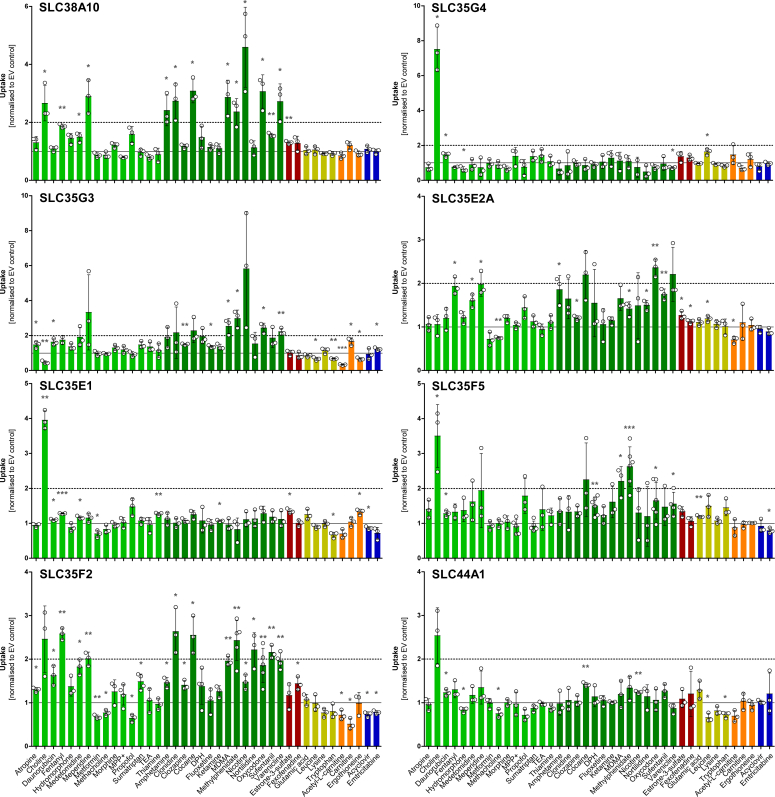
**Uptake of substances by selected orphan transporters.** HEK293 cells overexpressing a particular transporter and empty vector (EV)-transfected control cells were incubated with 2.5 μM (except nicotine with 10 μM) substrate for 2 min, and intracellular substrate concentration was quantified by HPLC-MS/MS analysis. Uptake is expressed as fold-increase in transporter-overexpressing cells over EV-transfected control, and individual data points are represented as mean ± SD of at least three independent experiments (Student’s *t* test; ∗*p* < 0.05, ∗∗*p* < 0.01, ∗∗∗*p* < 0.001). Substances include cations (*green*), known H^+^/OC antiporter substrates (*dark green*), anions (*red*), amino acids (*yellow*), zwitterions (*orange*), and nucleosides (*blue*). The *dashed horizontal lines* at 2.0 indicate the transport ratio above which the transport is generally considered medically relevant.

**Figure 2:**
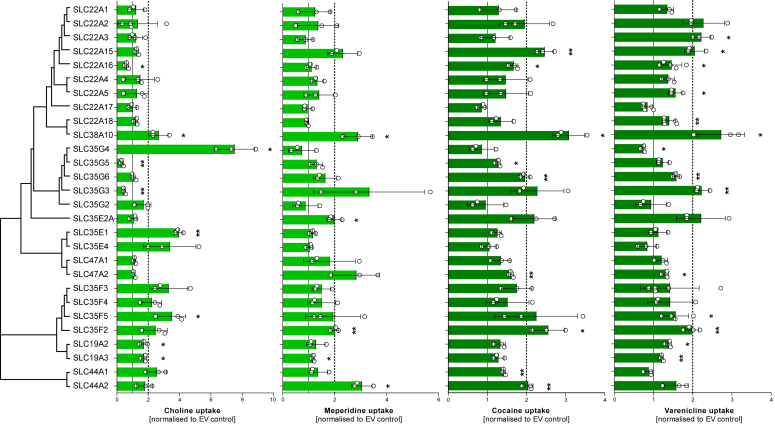
**Uptake of choline, meperidine, cocaine, and varenicline by known and orphan SLCs.** The cations choline and meperidine, as well as the H^+^/OC antiporter substrates cocaine and varenicline were incubated with HEK293 cells overexpressing a particular transporter and empty vector (EV)-transfected control cells at a concentration of 2.5 μM for 2 min, and intracellular substrate concentration was quantified by HPLC-MS/MS analysis. Uptake is expressed as fold-increase in transporter-overexpressing cells over EV-transfected control, and individual data points are represented as mean ± SD of at least three independent experiments (Student’s *t* test; ∗*p* < 0.05, ∗∗*p* < 0.01, ∗∗∗*p* < 0.001). The transporters are arranged by the protein similarities as determined with MUSCLE algorithm using Geneious Prime 2023.0.1.

The influx activity of all 28 cell lines was tested with 40 substrates under the same conditions using a 2.5 μM substrate concentration with an incubation time of 2 min. As illustrated in Figure 1 for selected eight SLCs, a significantly different pattern of substrate selectivity between the cell lines was found. Six of these transporters stood out as choline (cell influx) transporters with uptake ratios above the ratio of 2, which can be considered clinically relevant as suggested by FDA guidelines (https://www.fda.gov/media/134582/download). This enhancement of choline influx was already known for the choline transporter-like SLC44A1. Most other transporters with choline influx activity also had a moderate but statistically significant influx activity for numerous other organic cations. However, comparing the ratio of choline influx to the influx of the 39 other substrates, the transporters SLC35G4, SLC35F5, SLC35E1, and possibly the known choline-transporter-like SLC44A1 were relatively choline-specific (Fig. 2). Typically discussed substrates of the proton organic-cation antiporter (14) are highlighted in dark green in Figure 1. As judged from uptake ratios near to or above 2.0, five of these transporters had substrate spectra overlapping with the H^+^/OC antiporter, particularly SLC38A10 and SLC35F2.

To expand the range of biochemical properties in the search for substrates of the SLCs, organic anions, zwitterions, amino acids, and nucleosides were tested but only with a narrow spectrum of substrates (red for anions, yellow for amino acids, orange for zwitterions, and blue for nucleosides in Fig. 1). However, no substantial transport activities with these substrates were found. Apparently, in some substrate-transporter combinations, a significant efflux transport was found. These efflux activities not further analyzed here may be due to interactions with constitutively expressed transporters in the HEK293 cell line or interactions with endogenous substrates. For choline, carnitine, and the four amino acids glutamate, leucine, lysine, and tryptophan, we tried to reduce the latter interference by using isotope-labeled substrates, but acetylcarnitine, ergothioneine, and thiamine were not labeled.

In conclusion, six of these transporters had significant choline uptake activities, and four of the transporters (SLC38A10, SLC35E2A, SLC35F2, and -F5) had a broad, although only moderate, uptake capacity for numerous other organic cations at the substrate concentration of 2.5 μM. The uptake of four substrates by the 28 transporters, choline, meperidine, cocaine, and varenicline, is illustrated in comparison in Figure 2. The latter two are typical substrates of the genetically or as protein not yet identified H^+^/OC antiporter. Here, the increased uptake by SLC38A10, SLC35F2, and SLC35G3 is emphasized in more detail. As already highlighted above, SLC35G4 especially stood out as a choline transporter, but also some other SLCs, such as SLC38A10, SLC35E1, and SLC35F5, had statistically significant choline transport activity.

**Figure 3:**
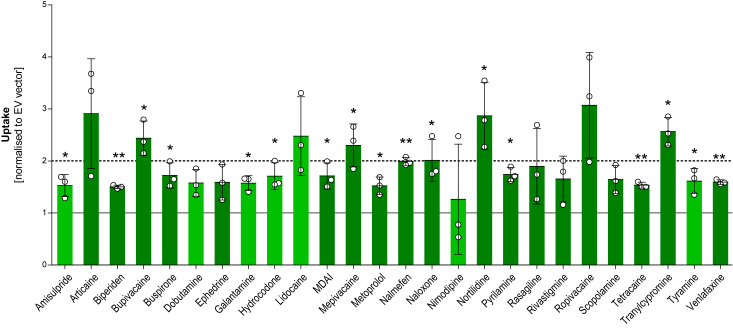
**Uptake of further cations and H**^**+**^**/OC antiporter substrates by SLC35G3.** Transport data of 26 cations identified as substrates of SLC35G3. Substrates were incubated with HEK293 cells overexpressing a particular transporter and empty vector (EV)-transfected control cells at a concentration of 2.5 μM for 2 min, and intracellular substrate concentration was quantified by HPLC-MS/MS analysis. Uptake is expressed as fold-increase in transporter-overexpressing cells over EV-transfected control, and individual data points are represented as mean ± SD of three independent experiments (Student’s *t* test; ∗*p* < 0.05, ∗∗*p* < 0.01, ∗∗∗*p* < 0.001). Known substrates of the H^+^/OC antiporter substrates are indicated in *dark green*.

Since SLC35G3 appeared in the first screening as a transporter with activities towards several organic cations, an extended substrate spectrum was tested with this SLC. As illustrated in Figure 3, the uptake of an additional 26 organic cations was significantly enhanced by this transport protein with uptake ratios between 1.5 and 3. Transport of other 76 substrates was not enhanced (Table S1). However, no clear molecular predictors could be identified differentiating SLC35G3 substrates from nonsubstrates.

**Figure 4:**
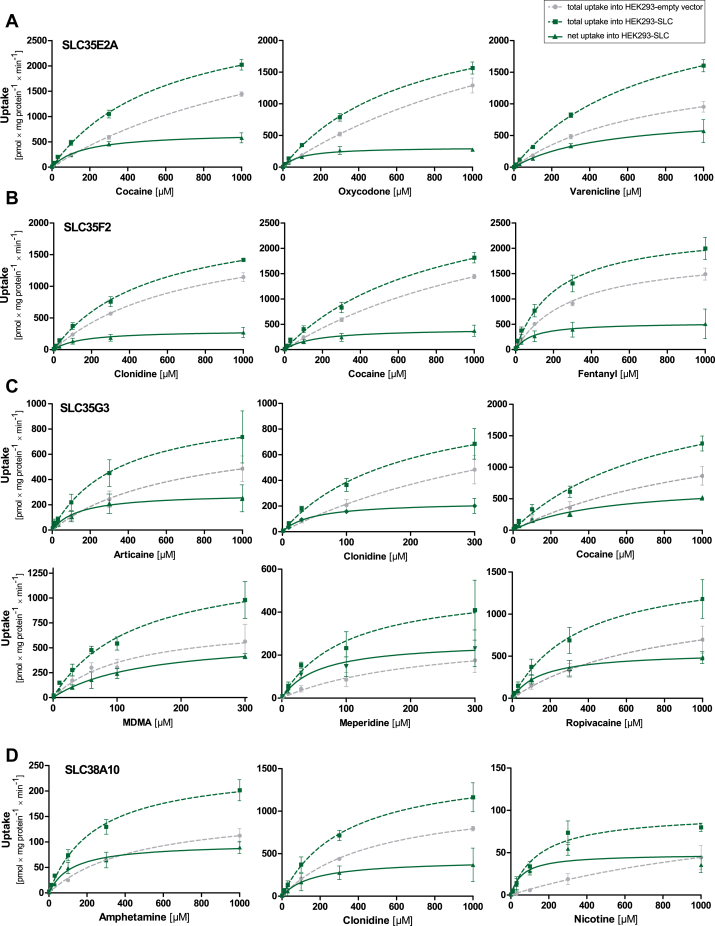
**Concentration-dependent transport of selected substances.** Concentration-dependent uptake of cationic substrates by HEK293 cells: panel (*A*) SLC35E2A, (*B*) SLC35F2, (*C*) SLC35G3, (*D*) SLC38A10. The *green dashed* curves indicate the total uptake by the cells overexpressing the respective recombinant SLC, and the *gray* lines indicate the uptake by the empty vector–transfected cells. The straight *green* curve shows the net uptake by the respective overexpressed transporter. Data are displayed as mean ± SEM of three independent experiments.

Based on the uptake ratios, carrier-mediated concentration-dependent uptake was studied with several pairs of cations and transporters to determine the kinetic parameters using the Michaelis–Menten model. As illustrated in Figure 4, most tested substrates displayed a saturable transport by the particular transporter. The transport kinetic parameters are listed in Table 2. By setting an intrinsic clearance of 3 as the cut-off for efficient transport, cocaine is significantly transported by SLC35E2A, fentanyl by SLC35F2, and clonidine, 3,4-methylenedioxymethamphetamine (MDMA; ecstasy), and meperidine are substrates for SLC35G3 which might play a pharmacokinetically significant role depending on the not yet fully known tissue expression of this SLC. As illustrated in Figure S2, substrates of several of the SLC35 transporters were relatively hydrophobic (compared to OCT1 substrates) and had a relatively small topological polar surface area. A small topological polar surface area is a prerequisite for good penetration through the blood–brain barrier (23).

**Table 2:** Kinetic parameters of the transport of selected substances

Transporter	Substance	V_max_ (±SEM) [pmol × mg protein^-1^ × min^-1^]	K_M_ (±SEM) [μM]	Cl_int_ (±SEM) [μl × mg protein^-1^ × min^-1^]
SLC35E1	Choline	87.1 (±14.5)	91.6 (±39.8)	0.95 (±0.57)
SLC35E2A	Cocaine	683 (±64.8)	165 (±47.7)	**4.14** (±1.59)
	Oxycodone	317 (±30.3)	92.0 (±31.0)	**3.44** (±1.49)
	Varenicline	847 (±163)	474 (±190)	1.79 (±1.06)
SLC35E4	Choline	41.9 (±30.3)	245 (±331)	0.17 (±0.36)
SLC35F2	Choline	81.0 (±11.0)	32.6 (±14.9)	2.48 (±1.47)
	Clonidine	304 (±57.7)	147 (±87.8)	2.06 (±1.62)
	Cocaine	415 (±83.5)	161 (±99.5)	2.58 (±2.11)
	Fentanyl	534 (±148)	90.1 (±26.8)	**5.92** (±3.41)
SLC35F3	Choline	135 (±34.8)	112 (±96.4)	1.21 (±1.36)
SLC35F4	Choline	62.8 (±18.9)	47.4 (±48.1)	1.32 (±1.74)
SLC35F5	Choline	34.8 (±12.1)	5.83 (±3.23)	**5.97** (±5.38)
SLC35G3	Articaine	291 (±76.8)	143 (±119)	2.04 (±2.24)
	Clonidine	234 (±36.9)	48.2 (±23.9)	**4.85** (±3.17)
	Cocaine	739 (±82.2)	466 (±117)	1.59 (±0.57)
	MDMA	606 (±102)	141 (±50.4)	**4.31** (±2.26)
	Meperidine	265 (±69.0)	57.7 (±44.9)	**4.60** (±4.78)
	Ropivacaine	551 (±70.9)	151 (±60.7)	**3.64** (±1.93)
SLC35G4	Choline	108 (±15.7)	13.2 (±7.02)	**8.15** (±5.5)
SLC38A10	Amphetamine	97.4 (±11.1)	120 (±44.9)	0.82 (±0.4)
	Clonidine	427 (±121)	162 (±141)	2.64 (±3.0)
	Nicotine	48.4 (±7.26)	62.0 (±35.6)	0.78 (±0.57)
SLC44A1	Choline	60.1 (±13.1)	28.9 (±18.5)	2.08 (±1.79)

Cl_int_, intrinsic clearance (intrinsic clearance values above 3 marked in bold characters); SEM, standard error of the mean parameter estimates based on at least three independent experiments.

**Figure 5:**
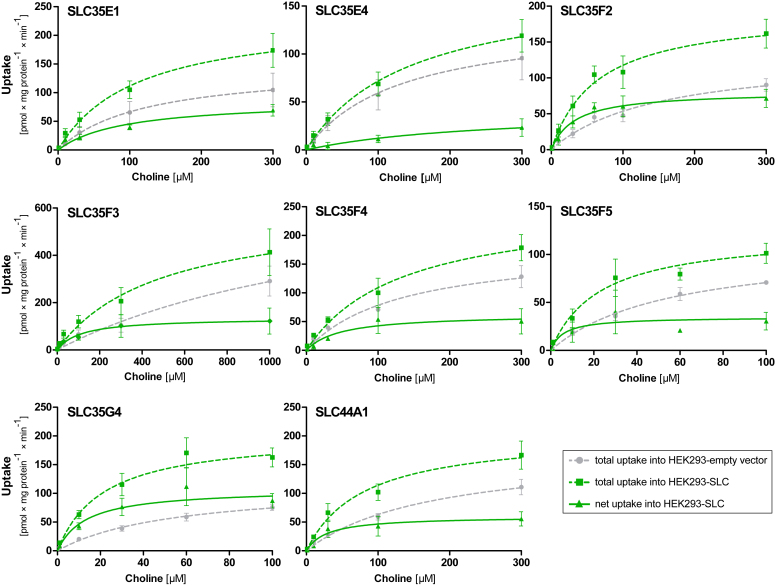
**Concentration-dependent uptake of choline by several SLCs.** Concentration-dependent uptake of deuterated (d9) choline by the HEK293 cells transfected with empty vector (*gray dashed* lines) and cells overexpressing the respective SLC (*green dashed* lines). Data are displayed as mean ± SEM of three independent experiments. As illustrated by the nonlinear concentration-uptake relationship in the empty vector–transfected cells, a significant endogenous choline transport activity is expressed in the HEK293 cells.

Choline was a substrate of eight out of the 28 transporters analyzed here. It displayed efficient carrier-mediated uptake by SLC35G4 as quantified by an intrinsic clearance of 8.15 μl/mg protein/min. The corresponding intrinsic choline clearance by SLC35F5 was 5.97, which is also illustrated by a saturable net uptake curve (Fig. 5*,* Table 2). Interestingly, SLC38A10 showed a noticeable saturable net uptake for nicotine. However, the kinetic analysis indicates a low intrinsic clearance due to a small V_max_ value and a high K_M_ value. This transport kinetic characteristics with a relatively small V_max_ and K_M_ were seen for several substances. As illustrated in Figures 4 and 5, for several substrates, a saturable (not linear) uptake into the control cell line suggests that those substances were in addition transported by other transport proteins constitutively expressed in the HEK293 cell line. Among other substances, this was always the case for choline, probably best explained by the fact that choline uptake is essential for the survival of the HEK293 cells.

One main aim of the present study was to investigate the identity of H^+^/OC antiporter activities, which can thus far not be attributed to specific genes and proteins (13, 14, 24). A characteristic feature of that H^+^/OC antiporter is its high activity in catalyzing the exchange transport of two organic cations. Therefore, we tested the antiport with diphenhydramine (DPH) and nalmefene. After preloading the hCMEC/D3 cell line with DPH, approximately 60% of the two substrates were exchanged within 2 min (unpublished data). With this pair of organic cations, several transporters from the SLC35 family (SLC35G4, SLC35E1, SLC35E4, and SLC35F4) showed a significant antiport with ratios as low as 0.7 (Fig. S3). Also, SLC22A15, -A6, and -44A1 catalyzed antiport with that pair of substrates. However, none of the overexpressed transporter’s antiport was as active as observed in the hCMEC/D3 cell line.

### Summary of the results

**Figure 6:**
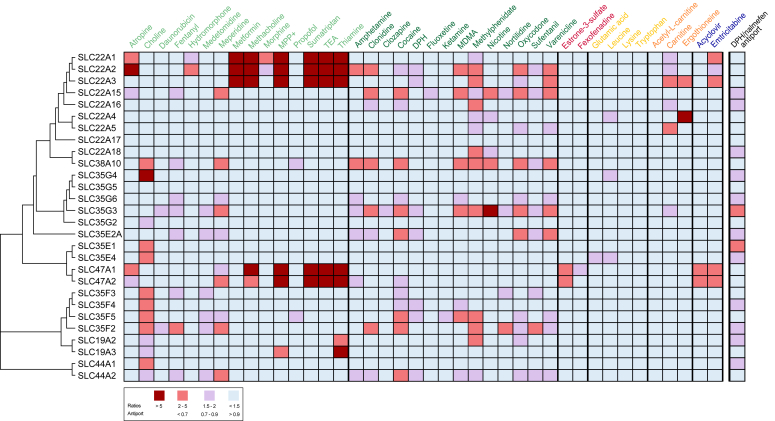
**Summary of uptake ratios for all investigated 28 solute carriers.** Uptake ratios of the 40 substrates by 28 transporters are shown in *dark red* for uptake ratios > 5, *light red* for 2 to 5, *light purple* for 1.5 to 2, and *light blue* for < 1.5. In this graph, uptake ratios between 1.0 and 1.5, even if statistically significant, are not shown as transport. The transporters are organized according to their sequence homology (MUSCLE 5.1 algorithm, Geneious Prime 2023.0.1). DPH, diphenhydramine; MDMA, 3,4-methylenedioxymethamphetamine; MPP^+^, 1-methyl-4-phenylpyridinium; TEA, tetraethylammonium. Numeric uptake data is provided in Table S2.

All data on the uptake of 40 test compounds by 28 SLCs are summarized in Figure 6. Here, in contrast to Figures 1 and 2, transport activities below a ratio of 1.5 were considered irrelevant, even if they were statistically significant. As in the figures above, transporters are ordered according to their sequence homology. The corresponding uptake data is listed in Table S2.

Fig. S4 presents an alternative clustering according to the similarities in the substrate spectrum. The pattern of substrate specificity seen there indicates that there are at least three distinct clusters. In addition, with the 40 substrates applied here, SLC22A2 (OCT2) appeared to have a distinct substrate preference, combining cluster 1 and cluster 4 activities.

Only two of the SLCs, SLC22A17 and SLC35G5, did not exhibit any transport activity with the 40 substrates and also showed no antiport activity (Figs. 6, and S3). A third one, SLC35G2, only showed weakly enhanced uptake of choline.

With the cutoff used here, the choline transporter-like (CTL) SLC44A1 and SLC35G4 appeared as selective choline transporter. A lower but also selective choline transport activity could be observed for SLC35E1 and -E4. In addition, several other members of the SLC35F family exhibited a relevant choline uptake activity, such as SLC35F3, -F4, and -F5, but they were not selective for choline as the only substrate. This substrate pattern was also seen for SLC38A10 and SLC44A2.

We tested several substrates that were earlier characterized as typical substrates of the H^+^/OC antiporter (columns 19–29 in Fig. 6) (14). Many of these antiporter substrates showed uptake by several transporters, such as SLC38A10, SLC22A15, SLC35G3, SLC35F2, and SLC35F5, but also SLC22A2 (OCT2) and SLC22A3 (OCT3). Several cationic substrates, such as tetraethylammonium (TEA), 1-methyl-4-phenylpyridinium (MPP+), and sumatriptan, of the well-characterized transporters SLC22A1, -2, and -3 and SLC47A1 and -2 could be confirmed as substrates of these transporters but were not transported by most of the newly identified organic cation transporters. That distinct substrate spectrum is also most evident from Fig. S4, where we see a distinct cluster 1 for the SLC22A1 to -A3 and the MATE transporters and a cluster of SLC22A15, SLC38A10, and some of the SLC35 family transporters.

Interestingly, but according to data already described before, a prototypic substrate of anion transporters, estrone-3-sulfate was confirmed to be transported by both SLC47A1 and -A2. Similarly, the two nucleoside analogs, acyclovir and emtricitabine, were only transported by the MATEs and only emtricitabine to some extent by OCT1 and -2, but not by the other transporters overexpressed here (Fig. 6).

The transporters could be clustered into several groups based on their substrate specificity: four were exclusive or predominant choline transporters, five had an OCT1-like substrate spectrum, and eight had a substrate spectrum similar to the spectrum of the H^+^/OC antiporter. Most of the latter group also had some choline transport activity, but the OCT1-like spectrum transporters (OCT1, -2, -3, and MATE1 and -2K) did not transport choline (Fig. S4).

As illustrated in Figures 1 and 2, for several pairs of transporters and substrates within the 2 min of transport measurement, there was no influx but a net efflux best explained by influx *via* constitutively expressed transporters or not transporter-mediated nonionic diffusion and efflux enhanced by the respective transporters. This efflux is also illustrated for several transporters in Fig. S5 as a negative correlation (Spearman’s rank correlation analysis) in the substrate spectra between some of the transporters (marked there in blue). Whereas substrates correlate well within the family SLC22A1-3 or SLC47A1 and -2, interestingly, the SLC35G family is not consistent in this analysis: substrates correlate well between SLC35G3, -5, and -6, but SLC35G2 or -4 do not show these similarities with them, although, the latter correlate well with each other.

## Discussion

Here, we comparatively characterized the membrane transport of 40 molecules by 28 SLCs. Although 18 of them were not identified as organic cation transporters before, 13 significantly enhanced cell influx transport of one or several organic cations. Significant influx transport of organic cations was mediated by SLC families thus far not associated with organic cation transport, including members of the SLC35 families G, E, and F and the transporter SLC38A10. As indicated in Table 1, some of the highly homologous transporters were at the same genomic location and most likely resulted from unequal crossover, but several other closely related transporters were on different chromosomes and probably originated from retrotransposons. In this project, we aimed to identify the genetically thus far uncharacterized proton/organic cation (H^+^/OC) antiporter, and, indeed, the substrate spectrum of several of the SLCs (particularly SLC22A15, -22A16, -38A10, -35G3, -35E2A, -35F2, and -35F5) resembled the substrate spectrum of the H^+^/OC antiporter (25, 26). Whether or not organic cation transport is a central function of these newly identified transporters or only a side-activity of other physiologically relevant transport functions cannot be decided based on the present data. As illustrated in Figure 1, Figure 2 and 6, the transport of several organic cations was enhanced only by a factor of about 2 and this was much lower than the transport of some substrates by OCT1, -2, and -3 or MATE1 and MATE2K shown for comparison here. However, these highly transported substrates of the well-known organic cation transporters were selected as excellent substrates based on an earlier screening of several hundreds of substrates. More extensive screening for cationic substrates of the newly identified organic cation transporters might also reveal substrates with higher transport rates. Future research might include both natural endogenous or exogenous substrates but also drugs and other synthetic substances.

Below, we briefly summarize existing and newly gained knowledge on the 28 SLCs studied. Concerning the group of less well-characterized orphan transporters, one or several genomic associations with cancer or other diseases are published for almost all of these genes. However, we commented on this data only if it could give a clue to the understanding of the transport functions of these SLCs. Several already well-characterized cation transporters (OCT1, -2, and 3, OCTN1 and -2, MATE1 and -2K) (27) were included mainly as a comparative scale for the new or less well-characterized organic cation transporters. Those seven well-characterized transporters have been proven to be expressed at the plasma membrane. For the orphan transporters, a cellular localization at the plasma membrane was indicated by bioinformatic prediction. However, for some of the transporters, additional expression in the mitochondria or Golgi apparatus is suggested, meaning they might have intracellular functions and transport functions at the outer cell membrane. Here, we have seen transport activity for most SLC, indicating that they are localized at least partly in the outer plasma membrane.

The gene coding for the first organic cation transporter, OCT1, was identified 30 years ago (2) and since then, the number of synthetic and natural substrates of OCT1 has increased to more than 300 (5, 7, 28). Typical substrates of OCT1 are positively charged at pH 7.4, relatively hydrophilic with a logD below 1.5 and have a molecular weight below 600 Da. However, also uncharged or negatively charged substances have been described as substrates (8). OCT1 is localized in a gene cluster with OCT2 and OCT3 on chromosome 6. As confirmed again in this study (Fig. 6), those three OCTs have several substrates in common, but there are also substrates only transported by one of the three. Also, tissue localization differs between them: whereas OCT1 is mostly hepatically and OCT2 mostly renally expressed, OCT3 is more ubiquitously expressed in several tissues, such as in the heart and the brain, as well as at the blood–brain barrier (10, 12, 29). Substrate polyspecificity is a characteristic of all three OCTs, which might be explained by different binding sites within the transporter (30, 31). Most recently, cryo-EM successfully elucidated OCT protein structures with high resolution and several substrates (3, 4, 32, 33, 34).

A further member of the SLC22 family is SLC22A15, which was identified as a polyspecific transporter of several organic cations and zwitterions (35). Overexpression of SLC22A15 resulted in extensive alterations in endogenous metabolomics (35). In this study, we did not measure significant uptake of carnitine and ergothioneine, but this may be due to different experimental conditions. SLC22A15 is highly expressed in several brain tissues, and we found that SLC22A15 significantly increased the uptake of meperidine, clonidine, cocaine, MDMA, nicotine, oxycodone, and varenicline. Considering its antiporter activity and expression in hCMEC/D3 cells, SLC22A15 may be one gene coding for the H^+^/OC antiporter.

SLC22A16 showed significantly increased and concentration-dependent uptake of tetraethylammonium, doxorubicin, and bleomycin (36, 37). However, measurements of anthracycline uptake by SLC22A16 in our laboratory found only minor enhancement of uptake below the margin of the uptake ratio of 2-fold (Redeker *et al.*, unpublished data). SLC22A16 may be interesting concerning individualized medicine (pharmacogenetics) because this gene has some frequent nonsynonymous polymorphisms.

The transporters SLC22A4 and -A5 can significantly enhance the membrane transport of some zwitterions. SLC22A4 or organic cation transporters novel 1 (OCTN1) was discovered as the ergothioneine transporter (38). Although minor to moderate transport activities for numerous others have been described (39), these activities were moderate only. SLC22A5 or OCTN2 was discovered as a high-affinity carnitine transporter more than 25 years ago, and a recessive inherited disease, primary systemic carnitine deficiency, can be caused by loss-of-function mutations in OCTN2 (40, 41). Here, we focused on whether the ubiquitously expressed OCTN2 is also a carrier of other organic cations. Numerous studies showed interactions with and the transport of cationic drugs *via* OCTN2, but many published transport rates were very low (1.1– 1.5-fold over empty-vector–transfected cells). This published transport data aligns with the findings here, where only minor to moderate transport rates were observed (Fig. 6).

In contrast to the rest of the SLC22A subfamily, SLC22A17 is not considered a transporter but as lipocalin-2 receptor (42). We included it in the screening because of its high homology to the other SLC22s. We found no transport activity (Fig. 6), although the gene was correctly genomically integrated, and mRNA was well expressed. Based on its strong homology to other SLCs, one might speculate whether it gains transport activity by binding lipocalin-2, but there is no experimental verification for that hypothesis.

The last member of the SLC22 family we investigated is SLC22A18 described in nearly 80 publications as a modulator of cell growth and other cell functions. However, thus far, no transport functions or substrates have been identified except for chloroquine and quinidine. However, this transport data was obtained by overexpression in bacteria and toxicity measurement (43). The earlier described biological functions on cell growth cannot be explained by its newly identified substrates, nicotine and methylphenidate. Thus, further studies, including broader metabolomics, still must be performed to identify the endogenous substrates possibly mediating its biological functions.

A protein with a particular structure differing from the other SLCs shown here is SLC38A10. It is extensively long with 1119 amino acids explained by four large intracellular and extracellular loops between the transmembrane domains. It was characterized as a transporter of aspartate and glutamate (44). A minor uptake of glutamic acid (by a factor of 1.4) compared with empty-vector–transfected cells was also seen in our screening but fell below our factor 1.5 cut-off. However, as shown in Figure 6, SLC38A10 has a broad substrate specificity towards numerous organic cations.

Like the SLC22 family, the SLC35 family is large and includes 31 genes. The subfamilies SLC35 A, B, C, and D have been identified as nucleotide sugar transporters (45), but that substrate specificity was not shown for the SLC35G subfamily, and the function of the subfamilies SLC35E, -F, and -G is still unclear. Many members of the SLC35G subfamily are expressed relatively broadly within the human body, but some are expressed mainly in the brain and the testicles (Table 1).

SLC35G4 has also been termed AMAC1L1, but with either name, we could not find any functional data during our literature research. According to our analyses, this SLC extensively enhanced the cell uptake of choline (Figs. 5 and 6) and thus it might be considered a CTL protein. Although its affinity (K_M_ 13.2 μM) is lower than the high-affinity choline transporter SLC5A7 (K_M_ 2 μM) (46), it still might play a physiologically relevant role in choline uptake since transport activities relevant for human beings often do not need to have very low affinities (K_M_ values) depending on substrate concentrations. Particularly for drugs, membrane transporters with only moderate affinity may still be most relevant as shown, for example, with the drug sumatriptan and OCT1 (47). Besides choline uptake, only the uptake of leucine was moderately enhanced by SLC35G5.

This transporter has been associated with resistance to platinum-based chemotherapy in high-grade ovarian cancer (48). However, this does not allow us to conclude that this SLC transports inorganic or organic platinum compounds because it was based on three cases and two controls only. In contrast to other investigated SLCs, none of the 40 substrates applied here was transported by SLC35G5. The next transporter SLC35G6 transported seven organic cations and had a moderate antiporter activity with diphenhydramine and nalmefene (Figs. 6 and S3). SLC35G6 was identified as a susceptibility marker for migraine and endometriosis (49), but there are no independent replication studies and there is no strong implication of organic cation transport in the pathophysiology of these two diseases.

SLC35G3 has not been associated with any transport functions before. In humans, it has a major expression in the testes and only minor expression in skeletal muscles. Figure 1, Figure 3, and 4 illustrate that SLC35G3 has a broad transport polyspecificity for 35 organic cations and zwitterions. Due to expression in the brain, SLC35G2 is a promising potential candidate for the H^+^/OC antiporter (https://www.proteinatlas.org/ENSG00000168917-SLC35G2/tissue; accessed on May 2nd 2024) (50). However, no transport function has been reported before. In our screening, it only showed a moderately enhanced choline influx.

The SLC35E subfamily can be found in skeletal muscle as well as in the brain. In particular, SLC35E2 was found in fetal brain (51). SLC35E2A (or SLC35E2) was not characterized concerning transport functions before. However, other members of the SLC35 family are known as transporters of nucleoside-conjugated sugars. Here, an enhanced transport of 10 organic cations was found with relatively high activity for cocaine, oxycodone, and varenicline. Based on bioinformatics, SLC35E2A was classified as a pseudogene enabling antiporter activity (https://www.ncbi.nlm.nih.gov/gene/9906, accessed on May 2nd 2024) (52), and indeed, cells overexpressing SLC35E2A exhibited antiporter activity (Fig. S3). According to the AlphaFold predicted protein structure, the four transmembrane domain protein structure does also not appear like typical SLCs. Thus, further experiments are required to elucidate how SLC35E2 exerts its transport function. Formation of dimers or interaction with constitutively expressed other membrane proteins in the HEK293 cells may explain the apparent functions.

Another member of the family, SLC35E1, was described as a protein relevant for the development of the herpes simplex type 1 virus (53), but this does not preclude transport activity for small molecules; there is another example of a transporter (NTCP) being important for the infection with hepatitis B virus and in parallel, being a transporter of several small molecular substances (54). According to bioinformatics, it is a transporter with antiporter activity, but experimentally, thus far, no transport activities have been reported. In our screening, only choline uptake was significantly enhanced, showing low affinity uptake for choline (Figs. 1 and 3), but a significant antiport activity was also found. SLC35E4 was not associated with any transport activity thus far. Here, we found moderate influx transport activity for choline and minor for the acidic and neutral amino acids glutamate and leucine.

SLC47A1 was identified as the human ortholog of the bacterial multidrug and toxin extrusion protein MATE1 (55). It is broadly expressed in several tissues. The other transporter from that family, SLC47A2 is predominantly expressed as a splice variant termed MATE2K. It is highly expressed in the human kidney. As an H^+^/OC antiporter, the transport direction is dependent on the pH gradient and in the kidneys, it usually excretes into the more acidic urinary side. As illustrated in Figure 6, the broad substrate spectrum of the two SLC47 transporters includes organic cations and some organic anions and nucleoside derivatives. Compared with the OCT1, -2, and -3 transporters, MATE1 as well as MATE2K have partially overlapping but partially distinct substrate spectra. As illustrated, the broad substrate spectrum does not mean lack of specificity; only about 10% of all small molecular organic cations are substrates of one of the two MATE transporters. Here, we could confirm substrates, such as TEA and MPP^+^, and also show moderate uptake of estrone-3-sulfate and acyclovir (11).

SLC35F subfamily members are expressed in several organs, such as endocrine tissue, skeletal muscle, and the brain. SLC35F3 was identified as a thiamine transporter, and a variant in the gene (rs17514104) was linked with the risk for hypertension and erythrocyte thiamine content (56). However, our experimental system revealed no enhanced thiamine uptake, but the earlier used *E. coli*–based transport experiments were quite different, and the genetic associations were not unequivocally replicated (56). Because SLC35F3 is capable of transporting several organic cations (Fig. 6), these associations may be due to endogenous organic cations other than thiamine. Similarly, SLC35F4 was also suggested as a thiamine-related gene based on its 92% homology to SLC35F3 and based on population genetic analyses (57). However, there is no experimental proof that SLC35F4 is a thiamine transporter, and in our system, no enhanced thiamine transport was measured. Here, we found enhanced cell uptake of choline, diphenhydramine, cocaine, and methylphenidate.

The expression of SLC35F5 was associated with response to 5-fluorouracil (58), but there is no unequivocal evidence that SLC35F5 does transport this pyrimidine derivative or its metabolites. A frameshift mutation was considered a dominant factor causing microcephaly and other malformations in a single child (59), but that causal relationship is not unequivocal. Here, we found enhanced uptake of 11 organic cations. The highest uptake rates were found for choline, cocaine, MDMA, and methylphenidate. Kinetic analysis of choline uptake showed an efficient cell uptake which might classify SLC35F5 as a CTL protein (Fig. 5). SLC35F2 was identified as a carrier of the anti-cancer drug sepantronium (60), and there were several other studies linking SLC35F2 with the progression of or response to cancer. However, specific drug substrates or endogenous small molecular modulators were not identified or verified by transport experiments. We found that SLC35F2 may transport a surprisingly broad spectrum of organic cations and might even be denominated as an opioid cell uptake transporter (Figs. 1 and 6).

The high-affinity thiamine (vitamin B1) transporter SLC19A2 transports thiamine, and its deficiency is a monogenetic trait associated with severe brain dysfunction. Thus, SLC19A2 is a cation transporter essential for vital function. However, similar to its homolog SLC19A1 and the SLC6A2, -3, and -4 genes specific for high-affinity reuptake of monoamine neurotransmitters, these specific transporters may transport numerous other substances with similar molecular features (21). As shown here, SLC19A2 may also enhance cell uptake of choline, methylphenidate, and the opioid oxycodone (Fig. 6). SLC19A3 is another biologically essential high-affinity thiamine transporter. Recently, it was identified that this transporter may also transport pyridoxine and pyridoxamine, the electroneutral and positively charged forms of vitamin B6 (61, 62). Similarly to SLC19A2, SLC19A3 also transports choline and has a moderate MPP^+^ transport capacity.

The last two transporters investigated here are SLC44A1 and A2, the CTLs CTL1 and CTL2. The cellular expression was suggested to be mitochondrial but it was also found in the outer plasma membrane (63). Both may be ethanolamine transporters and may contribute to choline transport through the blood–brain barrier (64). Here, we observed moderate choline uptake for both CTL1 and CTL2. However, concentration-dependent uptake revealed a relatively high K_M_ and a low intrinsic clearance (Table 2) for SLC44A1, which might indicate that the primary physiological role is not the transport of choline but the transport of other substrates such as ethanolamine. In contrast, while CTL1 had no other influx transport activity of the 39 tested substrates, CTL2 showed minor to moderate transport of several antiporter substrates. However, with the pairs of organic cations, diphenhydramine/nalmefene, SLC44A1, but not SLC44A2, exhibited significant antiport activity.

A simple association between substrate specificity and organ expression is unlikely. Many of the SLC here identified as organic cation transporters are relatively broadly expressed in different tissues, as far it is known (Table 1). However, it can be observed that most transporters with quaternary ammonium substrates like MPP^+^ and TEA are expressed in the kidneys or liver. Interestingly, some of the SCLs transporting cocaine, MDMA, or methylphenidate were indeed expressed in the brain and may thus contribute to the blood-brain barrier transfer of such substances acting in the brain (Tables 1 and 3), but it remains to be elucidated what their endogenous substrates are.

**Table 3:** Investigated SLCs and their substrates

Name	Major substrates identified here	Known substrates (selected)	References
SLC22A1		MPP^+^, TEA, thiamine, fenoterol, sumatriptan, ipratropium, dobutamine	(5, 7, 27)
SLC22A2		MPP^+^, TEA, atropine, tryptamine, salbutamol, histamine, terbutaline	(7, 76, 77)
SLC22A3		MPP^+^, TEA, phenformin famotidine, salbutamol, benzyltriethylammonium	(7, 78)
SLC22A15	Meperidine, clonidine, cocaine, MDMA, nicotine, oxycodone, varenicline	Ergothioneine, carnitine, MPP^+^, carnosine, gabapentin	(35)
SLC22A16	Clonidine, cocaine, methylphenidate	Carnitine, tetraethylammonium, doxorubicin, bleomycin	(36, 37)
SLC22A4	Methylphenidate, nicotine, leucine	Carnitine, ergothioneine, gabapentin	(38, 39, 79)
SLC22A5	Methylphenidate, oxycodone, varenicline	Carnitine, acetyl-carnitine	(40, 80)
SLC22A17	None	None (lipocalcin-2 receptor)	(42)
SLC22A18	Methylphenidate, nicotine	Chloroquine, quinidine	(43)
SLC38A10	Choline, amphetamine, cocaine, fentanyl, meperidine, clonidine	Glutamine, glutamic acid, aspartic acid	(44)
SLC35G4	Choline	None	
SLC35G5	None	None	
SLC35G6	Fentanyl, meperidine, amphetamine, cocaine, MDMA, oxycodone, varenicline	None	
SLC35G3	Nicotine, meperidine, clonidine, cocaine, MDMA, methylphenidate, oxycodone, varenicline, and others	None	
SLC35G2	Choline	None	
SLC35E2A	Cocaine, oxycodone, varenicline, and others	None	
SLC35E1	Choline	None	
SLC35E4	Choline, glutamate, leucine	None	
SLC47A1	Atropine, methacholine, MPP^+^, sumatriptan, estron-3-sulfate, acyclovir, emtricitabine	TEA, MPP^+^, metformin, cimetidine, creatinine, estrone-3-sulfate	(11)
SLC47A2	Meperidine, Metformin, MPP^+^, sumatriptan, TEA, thiamine, amphetamine, cocaine, estron-3-sultate	TEA, MPP^+^, metformin, cimetidine, creatinine, estrone-3-sulfate	(11)
SLC35F3	Choline, hydromorphone, medetomidine, cocaine, nortilidine, sufentanil	Thiamine	(56)
SLC35F4	Choline, cocaine, diphenhydramine, MDMA	None	
SLC35F5	Choline, cocaine, MDMA, methylphenidate	None	
SLC35F2	Choline, fentanyl, meperidine, clonidine, cocaine, methylphenidate, nortilidine, sufentanil	Sepantronium	(60)
SLC19A2	Choline, methylphenidate, oxycodone	Thiamine	(16, 17)
SLC19A3	Choline, MPP^+^	Thiamine, pyridoxamine	(17, 61, 62)
SLC44A1	Choline	Choline, ethanolamine	(18, 63)
SLC44A2	Choline, cocaine, meperidine	Choline, ethanolamine	(63)

SLC22A1-3 as well as SLC47A1-2 show a rather large substrate spectrum. For organic cation transporters, a negatively charged amino acid within the translocation path is assumed to be responsible for substrate specificity. As already mentioned, this substrate polyspecificity might be explained by different binding sites within the transporter, meaning several negatively charged amino acids (3, 65). In contrast, several transporters, such as SLC19A2-3, SLC35G4, and SLC44A1, display a small substrate spectrum which is focused on the endogenous substances thiamine or choline. A cluster analysis shown in Fig. S4 groups the investigated transporters in several clusters: firstly, transporters with strong uptake of cations such as MPP^+^, TEA, or sumatriptan are SLC22A1-3 and SLC47A1-2; secondly, transporters with marginal uptake of any tested substrate or increased uptake of only one or few substances, such as choline for SLC35E1 and -E4, SLC35F3 and-F4, SLC35G4 and -G2, and SLC44A1 or ergothioneine and carnitine for SLC22A4 and -A5; thirdly, transporters with significantly increased uptake of brain-active drugs and several H^+^/OC antiporter substrates (14).

Analysis of sequence motifs of the investigated SLCs might help to understand similarities and differences in substrate specificity. For that purpose, we applied the multiple expectation maximization for motif elicitation algorithms (66). For instance, SLC22A18 had a different substrate spectrum compared with several other transporters of the SLC22A family (Fig. 6) and correspondingly, SLC22A18 did not show any motifs that were prominent in the rest of the SLC22A family (Fig. S6). For SLC22A1, -2, and 3, the similarities in the sequence motifs and the overlapping substrate spectrum correlate well. Although several members of the SLC35 families generally show a high motif overlap, the motifs, for instance, in SLC35G2 or SLC35F5 were quite different, corresponding with differences in the substrate spectrum (Fig. 6). However, such correlations are still preliminary since we do not yet know the complete substrate spectrum of the transporters. In addition, substrate specificity is unlikely to be well explained by amino acid sequence motifs, considering that interactions between residues in different helices are relevant for substrate binding and transport (3, 32, 33, 67).

One obvious limitation of the present study is the lack of detailed characterization of each of the studied orphan transporters. In particular, it remains to be elucidated how the 4-transmembrane domain transporter SLC35E2A exerts its apparent transport activity and what the function of the extensive extracellular and intracellular loops linking the transmembrane domains in SLC38A10 is. However, these are special issues not affecting the major conclusions of the present data on transport activities with 40 substrates.

In conclusion, in addition to the about 10 well-characterized organic cation transporters mostly known for two or three decades, about 18 other SLCs do have significant organic cation transport activities. Thus far, these additional genes were classified as orphan transporters or transporters of other types of substrates. It could be shown that several cations, such as choline, and most substrates of the genetically not yet identified H^+^/OC antiporter activity were moderatedly transported by several of the newly identified organic cation transporters. The screening presented here might also be applied to functionally different gene families, and characterization of the genes presented here will have to be completed by a broader screening of drugs, other exogenous substances, and the endogenous metabolome and, finally, by structural characterization of these transporters.

## Experimental procedures

### Substances

All test compounds used for the present study were purchased from Sigma-Aldrich, AppliChem, Toronto Research Chemicals, Santa Cruz Biotechnology, and Cayman Chemicals with purities of at least 95%. The following endogenous substances were used as deuterated analogs to differentiate their intracellular concentrations resulting from the 2-min of influx transport from endogenous concentrations of these substances: choline-d9, carnitine-d6, glutamate-d5, leucine-d10, lysine-d3, and tryptophan-d5.

### Generation of cell lines

Experiments were performed in HEK293 cells overexpressing the mentioned SLC genes, and the transport data were normalized to the concentrations in HEK293 cells stably transfected with the empty vector. The cell lines were generated *via* stable transfection using the recombinant site-specific Flp-In system (Thermo Fisher Scientific). Generation of several stably overexpressing cell lines has been described in detail earlier as follows: empty vector pcDNA5 and OCT1 (68); OCT2 and OCTN1 and -N2 (69), OCT3 (65), MATE1 and -2K (70), THTR-1 and -2 (71).

The complementary DNA (cDNA) of the remaining transporters was purchased from RESOLUTE Consortium & Giulio Superti-Furga (72) (Table S3) and recloned into a pcDNA5/FRT vector (Thermo Fisher Scientific) *via* restriction enzyme digestion cloning. The restriction-site introducing PCR was composed of 5 μl of 10 × KOD buffer (KOD Hot Start DNA Polymerase Kit; Merck), 5 μl of dNTPs (2 mM each; Thermo Fisher Scientific), 2 μl of MgSO_4_ (25 mM; Merck), 1.3 μl of each forward and reverse primer (10 μM) characteristic to the transporter which included restriction sites for NheI and NotI (Table S4), 1 μl of HotStart KOD polymerase, 1 μl od DNA template (100 ng/μl), and 33.4 μl double-distilled water. The PCR protocol started with 3 min at 95 °C, followed by 35 cycles of 95 °C for 30 s, 56 °C for 30 s (63 °C for SLC35G3 and SLC44A2; 66 °C for SLC38A10) and 72 °C for 2 min (2:45 min for SLC38A10), and finished with 72 °C for 10 min. After the separation of the PCR product on an agarose gel, it was extracted from the gel (Gel Extraction Kit; Qiagen). Then, the plasmid DNA (41 μl) was digested with 2 μl of each restriction enzyme NheI-HF and NotI-HF (20 U/μl) and 5 μl of rCutSmart buffer (New England Biolabs) for 2 h at 37 °C. Afterwards, additional 1 μl of each enzyme was added, and the mixture was incubated for another hour. The digested product was separated on an agarose gel and cleaned up, as explained before. The DNA of each specific transporter was then ligated into the pcDNA5/FRT vector with the T4 DNA ligase kit (New England Biolabs) consisting of a suitable amount of transporter and vector DNA (3:1 ratio), 2 μl of 10× ligase buffer, 1 μl of T4 DNA ligase, and double-distilled water to fill up a total volume of 20 μl. The mixture was incubated for 10 min at room temperature and then heated to 65 °C for 10 min for heat inactivation. Finally, the ligated product was dialyzed for 30 min and transformed into OneShot TOP10 electrocompetent *E. Coli* (Thermo Fisher Scientific) *via* electroporation. The sequence of the ORF was validated *via* Sanger sequencing.

In preparation for the stable transfection, approximately 10^6^ HEK293 T-REx cells were seeded on a 6-well plate 24 h in advance. The cells were cultured with Dulbecco's Modified Eagles Medium (DMEM) supplemented with 10% (v/v) fetal bovine serum (FBS), penicillin (100 U/ml), and streptomycin (100 μg/ml) (Thermo Fisher Scientific). Separately from each other, the transfection reagent FuGene6 (12 μl) was diluted in 100 μl pure DMEM as well as 0.4 μg plasmid DNA and 3.6 μg pOG44 helper plasmid, which were diluted in another tube of 100 μl DMEM and incubated for 5 min. After the addition of the DNA mixture to the diluted transfection reagent, the DNA-FuGene6 mixture was incubated for 15 min. After the cells were washed with DMEM medium only supplemented with 10% FBS, the DNA-FuGene6 mixture was added dropwise to 1.8 ml of this medium. After an overnight incubation, the transfection medium was removed and replaced by fresh cell culture medium supplemented with FBS, penicillin, and streptomycin. The next day, the cells were transferred to a 100 mm cell culture dish and incubated again for 24 h. Finally, a concentration of 300 μg/ml of the selection antibiotic hygromycin B (Thermo Fisher Scientific) was added to the cells, and they were incubated until single colonies could be selected after nine to 10 days. The colonies were transferred to 24-well plates with a lower concentration of hygromycin B of 50 μg/ml and cultured further until they filled a T25 flask (25 cm^2^ surface area; Thermo Fisher Scientific) with a confluency of 80%. At this point, samples for DNA and RNA isolation were collected for genomic validation of the generated cell lines.

The cell lines, that were tested for *mycoplasma* exemplarily, were free of *mycoplasma* contamination.

### Genomic validation of generated cell lines

**Figure 7:**
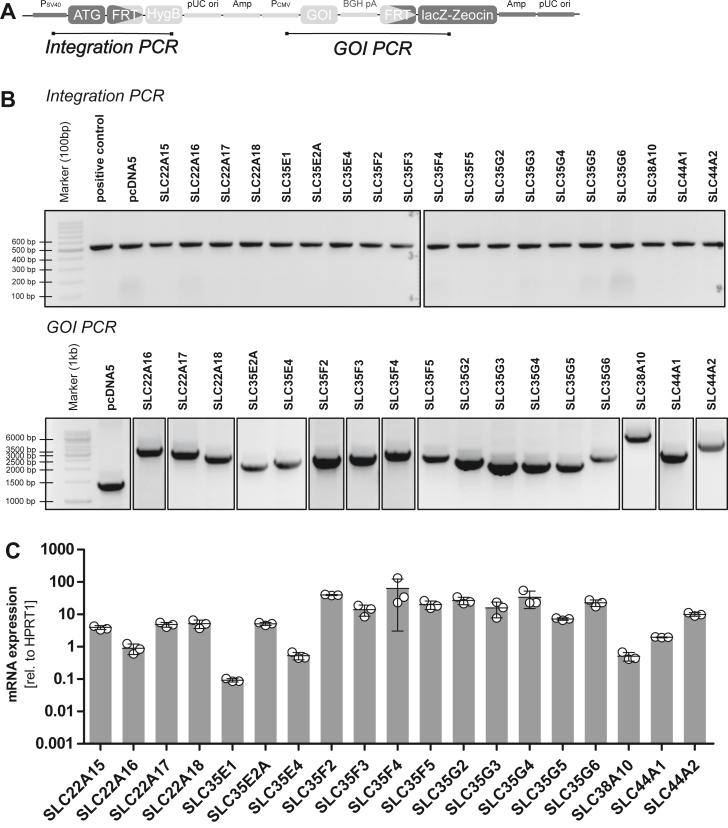
**Validation of generated cell lines.***A*, schematic view of integrated vector. The positions of PCR-amplified segments are marked. *B*, validation for correct integration by PCR. The results confirm the integration of the vector into the host genome and the presence of the gene of interest (GOI). The PCR products were separated at different times and on different gels; the splicing of the gel images is indicated with a *black* frame. *C*, gene expression analysis. The expression of mRNA was performed *via* real-time quantitative PCR normalized to the house-keeping gene HPRT1 for three different passages shown as mean ± SD (The GOI-PCR could not be performed for SLC22A15 and SLC35E1, but the mRNA expression for both transporters was detected).

The correct integration of the vector into the host genome was validated with an integration PCR that binds within the vector and the gene-of-interest PCR performed with gene-unspecific primers surrounding the gene locus (Fig. 7).

Firstly, ca. 2 × 10^6^ cells were harvested in an early passage and the genomic DNA was isolated using with QIAGEN DNeasy Blood and Tissue Kit (Qiagen). The QIAGEN Multiplex PCR Kit (Qiagen) was used for the integration PCR that consisted of 5 μl of 2 × QIAGEN Multiplex PCR master mix, 2 μl Q-solution, 0.25 μl of each forward and reverse primer (10 μM; Table S4), 1 μl of genomic DNA (100 ng/μl), and 1.5 μl double-distilled water. The PCR protocol started with 95 °C for min, followed by 35 cycles of 95 °C for 30 s, 62.7 °C for 1:30 min, and 72 °C for 1:30 min, and finished with 72 °C for 10 min. A correct integrated plasmid which had been validated previously was used as a positive control (519 bp).

The gene-of-interest PCR was performed with the Expand Long Template PCR system (Roche Diagnostics) that consisted of 2.8 μl of 10 × Expand Long buffer, 5.6 μl of Q-solution (Qiagen), 4.5 μl of dNTPs (each 2 mM; Thermo Fisher Scientific), 1.5 μl of MgSO_4_ (25 mM; Merck), 0.5 μl of each forward and reverse primer (10 μM; Table S4), 0.3 μl Expand Long polymerase mix, 3 μl DNA (80 ng/μl), and 9.3 μl double-distilled water. The PCR protocol started with 94 °C for 2 min, continued with 35 cycles of 96 °C for 10 s, 60 °C for 20 s, and 68 °C for 5 min, and ended with 68 °C for 7 min. The control for the gene-of-interest PCR was the empty pcDNA5/FRT vector cell line (1460 bp without the gene).

For analysis of PCR products, 1% (w/v) agarose gels supplemented with 0.5 μg/ml ethidium bromide were loaded with samples and connected to a voltage of approximately 120 V and a current below 80 mA to separate the PCR products in a mobile phase of 1× tris/borate/ethylenediaminetetraacetic acid supplemented with 0.5 μg/ml ethidium bromide. The DNA was visualized using the Fluor-S MultiImager (BioRad Laboratories) and the QuantityOne (version 4.2.3) software (Bio rad, https://www.bio-rad.com/de-de/product/quantity-one-1-d-analysis-software?ID=1de9eb3a-1eb5-4edb-82d2-68b91bf360fb).

### Quantification of gene expression

The quantification of gene expression was performed *via* quantitative real-time PCR.

At first, ca. 2 × 10^6^ cells were harvested from three early passages. After centrifugation at 400*g* for 4 min, the cell pellet was resuspended in 350 μl RLT lysis buffer with 1% beta-mercaptoethanol (v/v; Sigma-Aldrich). Then, the RNA was isolated using the QIAGEN RNeasy Plus Mini Kit (Qiagen). The cDNA synthesis was performed with the Superscript II Reverse Transcriptase Kit (Thermo Fisher Scientific). After dilution of 3 μg RNA in 17.75 μl RNase free water, 1 μl anchored dT primer (10 μM; 5′ -TTTTTTTTTTTTTTTTTTVN-3′; Thermo Fisher Scientific) was added and incubated at 70 °C for 10 min. Then, 11.25 μl of the reverse transcription mixture which contained 6 μl of 5× Superscript RT buffer, 3.5 μl of DTT (0.1 M), 1 μl of dNTPs (each 2 mM), 0.5 μl RNase inhibitor (40 U/μl), and 0.25 μl Superscript II Reverse Transcriptase (200 U/μl) was added and incubated at 42 °C for 1 h. Finally, the enzymes were denatured at 75 °C for 15 min, and the cDNA was diluted to 30 ng/μl with RNA-free water.

Quantitative real-time PCR (qPCR) was implemented with the HOT FIREPol EvaGreen qPCR Mix Plus Kit (Solis BioDyne, Tartu, Estonia) that consisted of 2 μl of 5× EvaGreen qPCR Mix, 0.4 μl of primer mixture (10 μM of each forward and reverse primer, Table S4), 5.6 μl double-distilled water, and 2 μl of 3 ng/μl cDNA. qPCR protocol was performed in technical triplicates in a 384-well plate using a Taqman 7900 T and analyzed with the SDS 1.2 software (Applied Biosystems, Themo Fisher Scientific, https://www.thermofisher.com/de/de/home/technical-resources/software-downloads/applied-biosystems-7500-real-time-pcr-system.html). The gene expression of the transporters was normalized to the housekeeping gene HPRT1. The cycle threshold values were evaluated using the following equation (73):

$$relative\;gene\;expression=2^{-\left({ct}_{WT}-{ct}_{WT,HPRT1}\right)}$$

### *In vitro* transport experiments

The HEK293 cells that were used for transport experiments were cultured in DMEM medium supplemented with 10% (v/v) FBS and the antibiotics penicillin (100 U/ml) and streptomycin (100 μg/ml).

In preparation for transport experiments, 24-well plates were pre-coated with poly-D-lysine (Sigma-Aldrich) and 300,000 cells were plated per well and incubated for 48 h. Firstly, the 24-well plate was placed on a heating plate at 37 °C, and the cells were washed with 37 °C HBSS^+^ buffer (Hank’s balanced salt solution supplemented with 10 mM Hepes and adjusted to pH 7.4; Thermo Fisher Scientific). The substance-of-interest, which was diluted in 37 °C HBSS^+^ to a desired concentration, was added to the cells for exactly 2 min. Transport ratios were performed with a substrate concentration of 2.5 μM, except for nicotine, which was tested with 10 μM because of mass spectrometric detection issues. The uptake was stopped with ice-cold HBSS^+^, and the 24-well plate was removed from the heating plate. The cells were washed twice with the cold HBSS^+^ before they were lysed for 15 min with 80% (v/v) acetonitrile containing the respective internal standard for mass spectrometry. For variations in seeded cell numbers between the cell lines, several wells were lysed in RIPA buffer and used for total protein quantification using a bicinchoninic assay (74) and bovine serum albumin as calibration.

For trans-stimulation experiments, cells were pre-loaded with 1 μM of diphenhydramine for 30 min at 37 °C before it was removed, and they were incubated with 250 μM of a second substance for 2 min as described above. As a control, cells were incubated with buffer for 2 min instead of another substance.

All *in vitro* transport experiments were performed at least three times in experiments performed on different days independently from each other.

### Concentration analysis *via* HPLC-MS/MS

The intracellular drug concentrations after transport experiments were analyzed by high-performance liquid chromatography coupled to tandem mass spectrometry analysis. The HPLC consisted of a Shimadzu Nexera HPLC System with a SIL-30AC autosampler, a LC-30AD pump, a CTO-20AC column oven, and a CBM-20A controller (Shimadzu). The reverse-phase chromatography of compounds was accomplished with a Brownlee SPP RP-Amide column with 4.6 × 100 mm inner dimensions, a particle size of 2.7 μm, and a preceding Phenomenex C-18 guard column. The mobile phase was composed of an organic additive with acetonitrile:methanol 6:1 (v/v) in total concentrations between 3% and 50% (v/v) and 0.1% (v/v) formic acid in the aqueous component of the eluent. During separation, the column oven was held at 40 °C and the mobile phase was maintained at a flow rate of 300 or 400 μl/min. After separation, the detection of substances was performed with an API 4000 tandem mass spectrometer (AB SCIEX, https://sciex.com/support/software-support/software-downloads), followed by quantification of results with the Analyst software (AB SCIEX, version 1.6.2). The composition of chromatographic conditions, as well as detailed mass spectrometric detection parameters, are listed in Table S5.

### Calculations

Transport-mediated net uptake was determined as fold-increase of transporter-overexpressing cells over empty vector–transfected control cells. Uptake ratios are displayed in bar graphs with individual data points represented as mean ± SD of three independent experiments (Student’s *t* test; ∗*p* < 0.05, ∗∗*p* < 0.01, ∗∗∗*p* < 0.001).

Kinetic parameters of concentration-dependent uptake experiments were determined with the net uptake of a transporter which was determined by the subtraction of total uptake into empty vector–transfected cells from total uptake into transporter-overexpressing cells. The K_M_ and V_max_ values were analyzed based on the Michaelis–Menten equation with *v* = *V*_max_ × [*S*]/(*K*_M_ + [*S*]) using GraphPad Prism (Version 5.01 for Windows, GraphPad Software, https://www.graphpad.com). While V_max_ describes the maximum transport velocity, K_M_ defines the substrate concentration required to reach half V_max_. The ratio of V_max_ over K_M_ is termed intrinsic clearance Cl_int_.

Biochemical parameters were calculated with the Instant JChem package and MarvinSketch of Chemaxon (version 21.2.0, Budapest, Hungary). For sequence homology analysis, Geneious Prime 2023.0.1 was used with the MUSCLE 5.1 algorithm (https://www.geneious.com). Analysis of sequence motifs reoccurring in the 28 investigated SLCs was performed with MEME (Multiple Em for Motif Elicitation) Suite 5.5.5, a motif-based sequence analysis tool.

## Data availability

All data supporting the conclusions of this article are presented in the main manuscript and in the supplementary data. Additional data will be made available by the authors on request.

## Supporting information

This article contains supporting information.

## Conflicts of interest

The authors declare that they have no conflicts of interest with the contents of this article.
