# Supplementary material for: Several orphan solute carriers functionally identified as organic cation transporters: Substrates specificity compared with known cation transporters
Source: J Biol Chem. 2024 Aug 3;300(9):107629. doi: 10.1016/j.jbc.2024.107629 (PMC11406361; doi:10.1016/j.jbc.2024.107629)
Supplement: Supporting Information [file mmc1.docx]

**Supporting information**

Title:

Several orphan solute carriers functionally identified as organic cation transporters: substrates specificity compared with known cation transporters

Authors: Kyra-Elisa Maria Redeker and Jürgen Brockmöller

Figure S1: Protein sequence alignment

Figure S2: Chemical properties of identified substrates

Figure S3: Trans-stimulated uptake of nalmefen in exchange with DPH

Figure S4: Cluster analysis of 28 SLC based on substrate similarities

Figure S5: Correlation of substrate specificity of 28 SLCs

Figure S6: Reoccurring sequence motifs in 28 SLCs

Table S1: Uptake data of 102 tested substances for SLC35G3

Table S2: Uptake data of 40 tested substances for 28 SLC

Table S3: cDNA of investigated SLCs

Table S4: Primers used for the generation and validation of orphan transporters

Table S5: HPLC-MS/MS parameter

**Figure S1:** Protein sequence alignment.

Sequence alignment of 26 of the 28 genes using the MUSCLE 5.1 algorithm (Geneious Prime 2023.0.1). Sequences of SLC35E2A and SLC38A10 are not shown because of their unique genetic architectures (4 transmembrane helices and very large interhelical loops, respectively).

**Figure S2:** Chemical properties of identified substrates.

The three chemical properties molecular weight, logD value at pH 7.4, and the topological polar surface area (TPSA) in comparison for substrates of the investigated SLCs (MarvinSketch Chemaxon 21.2.0, Budapest, Hungary).

**Figure S3:** Trans-stimulated uptake of nalmefen in exchange with DPH.

After pre-loading of HEK293 cells overexpressing a particular transporter with 1 µM DPH (diphenhydramine) for 30 min, the cells were incubated with 250 µM nalmefen for 2 min (control only with buffer). Exchange rate of DPH was normalized to a DPH control. Individual data points are represented as mean ± SD of three independent experiments (Student’s t-test; *p <0.05, **p <0.01, ***p <0.001). A trans-stimulation ratio < 0.7 indicating antiport is marked with a dotted line.

[Fig. S4 in landscape orientation]

**Figure S4:** Cluster analysis of 28 SLC based on substrate similarities.

Uptake ratios of overall 40 substances by 28 transporters from *Fig. 6* organized based on cluster analysis of substrate similarities. Transporter overexpressing HEK293 cells were incubated with 2.5 µM (exception: nicotine 10 µM) substrate for 2 min and normalized to empty vector-transfected control cells. Abbreviations: DPH, diphenhydramine; MDMA, 3,4-methylenedioxymethamphetamine; MPP^+^, 1-methyl-4-phenylpyridinium; TEA, tetraethylammonium.

**Figure S5:** Correlation of substrate specificity of 28 SLC.

Substrate correlation analysis (Spearman’s rank correlation) with correlation coefficient from -1 (blue) as negative correlation to 0 (white) to 1 (green) as positive correlation.

**Figure S6:** Reoccurring sequence motifs in 28 SLCs.

Analysis of sequence motifs reoccurring in the 28 investigated SLCs performed with MEME - Multiple Expectation-maximum for Motif Elicitation (MEME Suite 5.5.5). Details about the MEME (Multiple Expectation maximization for Motif Elicitation) algorithms are given by T.L. Bailey, Current Protocols in Bioinformatics (2003) 2.4.1-2.4.35 [Reference (66)].

**Table S1:** Uptake data of 102 tested substances for SLC35G3

| **Substance** | **Mean value** | **±SD** |
| --- | --- | --- |
| Acyclovir | 1.21 | 0.24 |
| Amiodaron | 1.35 | 0.32 |
| **Amisulpride** | **1.53** | **0.21** |
| Amitriptyline | 1.47 | 0.15 |
| Amphetamine | 1.28 | 0.39 |
| Aripiprazole | 1.22 | 0.18 |
| **Articaine** | **2.91** | **1.05** |
| Aspartame | 0.92 | 0.26 |
| Atenolol | 0.97 | 0.17 |
| Atomoxetine | 1.26 | 0.09 |
| Atropine | 1.04 | 0.19 |
| Betaine | 0.84 | 0.05 |
| **Biperiden** | **1.50** | **0.03** |
| Bisoprolol | 1.24 | 0.09 |
| **Bupivacaine** | **2.44** | **0.33** |
| Bupropion | 1.29 | 0.09 |
| **Buspirone** | **1.72** | **0.24** |
| Cabergoline | 1.49 | 0.29 |
| Carnitine-d9 | 1.05 | 0.10 |
| Cathine ((+)-Norpseudoephedrine) | 1.21 | 0.23 |
| Cetirizine | 1.21 | 0.35 |
| Clemastine | 1.22 | 0.04 |
| Clonidine | 1.54 | 0.18 |
| Clozapine | 1.44 | 0.10 |
| Cocaine | 2.02 | 0.39 |
| Cycloserine (D-) | 0.93 | 0.20 |
| Cycloserine (L-) | 1.11 | 0.11 |
| Desipramine | 1.31 | 0.12 |
| Dimenhydrinate | 1.37 | 0.80 |
| Diphenhydramine | 1.53 | 0.23 |
| **Dobutamine** | **1.58** | **0.26** |
| Doxycycline | 1.34 | 0.35 |
| Doxylamine | 1.10 | 0.57 |
| Duloxetine | 1.26 | 0.03 |
| **Ephedrine** | **1.59** | **0.34** |
| Ergothionine | 1.67 | 0.44 |
| Ethambutol | 0.99 | 0.14 |
| Fenoterol | 1.19 | 0.38 |
| Fluoxetine | 1.37 | 0.06 |
| Formoterol | 1.01 | 0.34 |
| Frovatriptan (R-) | 1.06 | 0.02 |
| Gabapentin | 0.90 | 0.13 |
| **Galantamine** | **1.57** | **0.14** |
| Haloperidol | 1.47 | 0.32 |
| **Hydrocodone** | **1.71** | **0.26** |
| Hydromorphone | 1.28 | 0.03 |
| Imipramine | 1.40 | 0.07 |
| Ipratropium | 1.25 | 0.20 |
| Ketamine | 1.28 | 0.17 |
| Levofloxacin | 1.05 | 0.28 |
| Levomepromazine | 1.30 | 0.13 |
| **Lidocaine** | **2.48** | **0.76** |
| **MDAI** | **1.71** | **0.25** |
| MDMA | 1.58 | 0.46 |
| Memantine | 1.19 | 0.25 |
| **Mepivacaine** | **2.30** | **0.41** |
| Meropenem | 1.27 | 0.28 |
| Mescaline | 1.34 | 0.04 |
| Methylnaltrexon | 1.36 | 0.41 |
| Methylphenidate | 1.31 | 0.60 |
| Metoclopramide | 1.44 | 0.27 |
| **Metoprolol** | **1.52** | **0.17** |
| Midodrine | 1.33 | 0.20 |
| Milnacipran | 1.31 | 0.12 |
| Morphine | 1.20 | 0.07 |
| Moxifloxacin | 1.34 | 0.58 |
| **Nalmefen** | **1.99** | **0.08** |
| **Naloxone** | **2.01** | **0.40** |
| N-Desmethyltramadol | 1.38 | 0.03 |
| N-Desmethylvenlafaxine | 1.20 | 0.13 |
| **Nimodipine** | **1.26** | **1.06** |
| **Nortilidine** | **2.87** | **0.64** |
| Oxycodone | 1.86 | 0.17 |
| Palonosetron | 1.36 | 0.10 |
| Paroxetin | 1.30 | 0.04 |
| Phenylephrine | 1.10 | 0.10 |
| Pipamperone | 1.33 | 0.10 |
| Propranolol | 1.27 | 0.17 |
| **Pyrilamine** | **1.74** | **0.14** |
| Quetiapine | 1.37 | 0.24 |
| **Rasagiline** | **1.90** | **0.73** |
| Risperidone | 1.41 | 0.18 |
| **Rivastigmine** | **1.65** | **0.44** |
| Ropinirole | 1.37 | 0.17 |
| **Ropivacaine** | **3.07** | **1.01** |
| **Scopolamine** | **1.64** | **0.27** |
| Solifenacin | 1.26 | 0.14 |
| Sufentanil | 1.72 | 0.17 |
| Tamsulosin | 1.39 | 0.04 |
| Terazosin | 1.49 | 0.27 |
| Terfenadine | 1.28 | 0.01 |
| **Tetracaine** | **1.54** | **0.06** |
| Thiamine | 0.90 | 0.42 |
| **Tranylcypromine** | **2.56** | **0.27** |
| Trimethoprim | 1.24 | 0.13 |
| Trimipramine | 1.47 | 0.38 |
| **Tyramine** | **1.61** | **0.24** |
| Urapidil | 1.37 | 0.30 |
| Varenicline | 1.60 | 0.16 |
| **Venlafaxine** | **1.60** | **0.04** |
| YM155 | 1.11 | 0.16 |
| Zolmitriptan | 1.27 | 0.20 |

Substances in bold characters are displayed in Fig. 3 of the main manuscript.

**Table S2:** Uptake data of 40 tested substances for 28 SLC

corresponding data to Fig. 1, 2 and 6 of the main manuscript.

**Table S3:** cDNA of investigated SLCs

| **Name** | **Addgene plasmid** | **Link** | **RRID** |
| --- | --- | --- | --- |
| pDONR221-SLC22A15_STOP | # 161177 | http://n2t.net/addgene:161177 | Addgene_161177 |
| pDONR221-SLC22A16_STOP | # 161189 | http://n2t.net/addgene:161189 | Addgene_161189 |
| pDONR221-SLC22A17_STOP | # 161201 | http://n2t.net/addgene:161201 | Addgene_161201 |
| pDONR221-SLC22A18_STOP | # 161213 | http://n2t.net/addgene:161213 | Addgene_161213 |
| pDONR221-SLC38A10_STOP | # 161249 | http://n2t.net/addgene:161249 | Addgene_161249 |
| pDONR221-SLC35G4_STOP | # 161286 | http://n2t.net/addgene:161286 | Addgene_161286 |
| pDONR221-SLC35G5_STOP | # 161274 | http://n2t.net/addgene:161274 | Addgene_161274 |
| pDONR221-SLC35G6_STOP | # 161262 | http://n2t.net/addgene:161262 | Addgene_161262 |
| pDONR221-SLC35G3_STOP | # 161298 | http://n2t.net/addgene:161298 | Addgene_161298 |
| pDONR221-SLC35G2_STOP | # 161310 | http://n2t.net/addgene:161310 | Addgene_161310 |
| pDONR221-SLC35E2A_STOP | # 161252 | http://n2t.net/addgene:161252 | Addgene_161252 |
| pDONR221-SLC35E1_STOP | # 161264 | http://n2t.net/addgene:161264 | Addgene_161264 |
| pDONR221-SLC35E4_STOP | # 161311 | http://n2t.net/addgene:161311 | Addgene_161311 |
| pDONR221-SLC35F3_STOP | # 161275 | http://n2t.net/addgene:161275 | Addgene_161275 |
| pDONR221-SLC35F4_STOP | # 161263 | http://n2t.net/addgene:161263 | Addgene_161263 |
| pDONR221-SLC35F5_STOP | # 161251 | http://n2t.net/addgene:161251 | Addgene_161251 |
| pDONR221-SLC35F2_STOP | # 161287 | http://n2t.net/addgene:161287 | Addgene_161287 |
| pDONR221-SLC44A1_STOP | # 161079 | http://n2t.net/addgene:161079 | Addgene_161079 |
| pDONR221-SLC44A2_STOP | # 161380 | http://n2t.net/addgene:161380 | Addgene_161380 |

**Table S4:** Primers used for the generation and validation of orphan transporters

| **Primers for restriction enzyme cloning into pcDNA5/FRT vector** | | | |
| --- | --- | --- | --- |
| Transporter | Direction | Sequence (5 🡪 3) with restriction sites NheI in forward and NotI in reverse | |
| SLC22A15 | forward  reverse | AAAAAGCAGCTAGCACC**ATG**GAGGTGGAGGAGGCA  GCTGGGTCGCGGCCGC**TTA**CTTGATCATCTGGGT | |
| SLC22A16 | forward  reverse | AAAAAGCAGCTAGCACC**ATG**GGCAGCCGCCACTTC  GCTGGGTCGCGGCCGC**TTA**CTCGCCCAGGCCGGA | |
| SLC22A17 | forward  reverse | AAAAAGCAGCTAGCACC**ATG**GCCTCCGACCCTATC  GCTGGGTCGCGGCCGC**TTA**CAGGGCTGGATTAGG | |
| SLC22A18 | forward  reverse | AAAAAGCAGCTAGCACC**ATG**CAGGGAGCAAGGGCA  GCTGGGTCGCGGCCGC**TTA**GCGCACCTTGTCCTTCC | |
| SLC38A10 | forward  reverse | AAAAAGCAGCTAGCACC**ATG**ACCGCAGCAGCAGCA  GCTGGGTCGCGGCCGC**TTA**GCTCTCCTCTGGAGGGC | |
| SLC35G4 | forward  reverse | AAAAAGCAGCTAGCACC**ATG**GCCGGCTCCCACCCTT  GCTGGGTCGCGGCCGC**TTA**CTCCTCCACCTTGCCTG | |
| SLC35G5 | forward  reverse | AAAAAGCAGCTAGCACC**ATG**GCCGGCAGCCACCCT  GCTGGGTCGCGGCCGC**TTA**CTCCTCCACCTTGCC | |
| SLC35G6 | forward  reverse | AAAAAGCAGCTAGCACC**ATG**GCAGGAAGCCACCCT  GCTGGGTCGCGGCCGC**TTA**CTCCTCCACCTTGCC | |
| SLC35G3 | forward  reverse | AAAAAGCAGCTAGCACC**ATG**GCCGGCAGCCACCCA  GCTGGGTCGCGGCCGC**TTA**CTCCTCCACGCGGCC | |
| SLC35G2 | forward  reverse | AAAAAGCAGCTAGCACC**ATG**GACACCAGCCCATCC  GCTGGGTCGCGGCCGC**TTA**CTTGATGGGGGAATCC | |
| SLC35E2A | forward  reverse | AAAAAGCAGCTAGCACC**ATG**AGCTCCTCTGTGAAG  GCTGGGTCGCGGCCGC**TTA**GGAGGCAGGCAGCAT | |
| SLC35E1 | forward  reverse | AAAAAGCAGCTAGCACC**ATG**GCAGCAGCAGCAGTG  GCTGGGTCGCGGCCGC**TTA**CACGTCATAGCGATTCA | |
| SLC35E4 | forward  reverse | AAAAAGCAGCTAGCACC**ATG**TGCAGGTGTCCACCTG  GCTGGGTCGCGGCCGC**TTA**CAGGCCCTTGCTTGGCT | |
| SLC35F3 | forward  reverse | AAAAAGCAGCTAGCACC**ATG**AAGAAGCACTCCGCCA  GCTGGGTCGCGGCCGC**TTA**TCTGGCAAAAGATGGCC | |
| SLC35F4 | forward  reverse | AAAAAGCAGCTAGCACC**ATG**GACGAGCTGCTGCTG  GCTGGGTCGCGGCCGC**TTA**GGCCAGAGGGATGCT | |
| SLC35F5 | forward  reverse | AAAAAGCAGCTAGCACC**ATG**GTGCCACCTCGGAGAC  GCTGGGTCGCGGCCGC**TTA**AGATGCTCCGTCCTCCT | |
| SLC35F2 | forward  reverse | AAAAAGCAGCTAGCACC**ATG**GAGGCCGATAGCCCTG  GCTGGGTCGCGGCCGC**TTA**CAGCACGGCGGAGTGGG | |
| SLC44A1 | forward  reverse | AAAAAGCAGCTAGCACC**ATG**GGATGCTGTAGCTCCG  GCTGGGTCGCGGCCGC**TTA**GGCGCTAGAGGCTCCGG | |
| SLC44A2 | forward  reverse | AAAAAGCAGCTAGCACC**ATG**GGCGACGAGCGGCCC  GCTGGGTCGCGGCCGC**TTA**AGACTCGGCGGCCTTCT | |
| **Primers for genomic validation** | | | |
| PCR | Primer | Sequence (5 🡪 3) | Amplicon size [bp] |
| Integration PCR | P_SV40_  P_Hyg_r2_ | AGCTGTGGAATGTGTGTCAGTTAGG  ACGCCCTCCTACATCGAAGCTGAAA | 519 |
| Gene-of-interest PCR | P_CMV_  P_LacZ_ | CCATGGTGATGCGGTTTTGGCAGTA  CCTTCCTGTAGCCAGCTTTCATCAA | pcDNA5/FRT control 1460 |
| **Primers for quantitative real-time PCR** | | | |
| Gene | Direction | Sequence (5 🡪 3) | Amplicon size [bp] |
| HPRT1 | forward  reverse | TGACACTGGCAAAACAATGCA  GGTCCTTTTCACCAGCAAGCT | 94 |
| SLC22A15 | forward  reverse | TCAGCAGTGCGTGGATAAGG  GGCGTCGTAAAACTCCTCCT | 70 |
| SLC22A16 | forward  reverse | GCCTTCGCCGTGGATTACTA  TCACATACACGAAGCCCACC | 94 |
| SLC22A17 | forward  reverse | GAACAGACCACACGGACAGA  GCAGTGGGCAGGTATTCTCC | 77 |
| SLC22A18 | forward  reverse | GCTCCGTGTTCCACTTTTGC  GGTATCGCTGGTAGACACGG | 110 |
| SLC38A10 | forward  reverse | CGTGATGGTGGGCTTCTTTG  GGAAGTGCATCAGCACGTTG | 71 |
| SLC35G4 | forward  reverse | AACCGGAAATGCAGCAACAG  CAGTCCGCACCAGTCATAGC | 109 |
| SLC35G5 | forward  reverse | GCCTATCAGGGCAGCAATCT  GCGATTGGCAGGTGAAACAG | 71 |
| SLC35G6 | forward  reverse | CAGGAAATGCAGCAACCGTG  CACCAGTCATAGCCGCTCAG | 100 |
| SLC35G3 | forward  reverse | TGTTTCACCTGCCTATCGCC  CGCAAAAGAAGGCTCTGCTC | 90 |
| SLC35G2 | forward  reverse | GAGATCGGCCAGTTCCAGTC  TCCAAACAGCACGATCCACA | 75 |
| SLC35E2A | forward  reverse | CCCTGCTGTACCTGACACTG  CAGCATGGATGGCTCTCCTC | 98 |
| SLC35E1 | forward  reverse | TTGACATGTGGGGACTGGTG  CCTCAGGTGGTGGATTCTGG | 107 |
| SLC35E4 | forward  reverse | TGCGGAAATGTGGGACTGAG  GACAGGGCCAGGGTAAACAG | 86 |
| SLC35F3 | forward  reverse | TTCTTTGGCGATAACGGCCT  AGTTTGTCAGGGTCCACAGC | 82 |
| SLC35F4 | forward  reverse | TATGGGCGTGAGAATCGTGG  GATGATGCTATCGGCGTGGA | 91 |
| SLC35F5 | forward  reverse | TCCCATCCAATTCTGGCGAC  AGCACCACTCCTCCGATAGA | 76 |
| SLC35F2 | forward  reverse | GCAGGAAGGGAGGACAACAG  GATGGCGTACAGGGATGCTC | 81 |
| SLC44A1 | forward  reverse | AGGACGCCTTTGTGATCCTG  CAGCATGAAATCGCCCACTG | 74 |
| SLC44A2 | forward  reverse | CATCCAGGGCAGTGGGATAC  TCAGAAACACGGCGGTAGAG | 108 |

**Table S5:** HPLC-MS/MS parameter

| Substance | RT  [min] | Q1 mass [Da] | Q3 mass  [Da] | DP  [V] | CE  [V] | CXP  [V] | Internal Standard |
| --- | --- | --- | --- | --- | --- | --- | --- |
| 3% organic additive (96.9% ddH_2_O, 0.1% formic acid, 2.6% acetonitrile, 0.4% methanol) | | | | | | | |
| Buformin | 4.1 | 158.0 | 60.0 | 40 | 35 | 10 |  |
| Carnitine-d9 | 2.9 | 171.3 | 85.0 (129.0) | 81 | 29 (9) | 6 (10) | Buformin |
| Choline-d9 | 2.9 | 113.108 | 69.1 (66.1) | 66 | 27 (44) | 12 | Buformin |
| Ergothioneine | 3.4 | 230.159 | 127.0 (186.2) | 50 | 25 (17) | 8 (12) | Buformin |
| Glutamate-d5 | 3.3 | 152.8 | 135.1 (88.1) | 36 | 13 (23) | 8 (16) | Buformin |
| Leucine-d10 | 4.2 | 142.1 | 96.2 (50.2) | 50 | 15 (36) | 18 (8) | Buformin |
| Lysine-d3 | 3.6 | 150.12 | 87.2 (133.1) | 31 | 22 (8) | 16 (8) | Buformin |
| Metformin | 2.9 | 130.0 | 71.0 | 40 | 35 | 10 | Buformin |
| Methacholine | 3.2 | 160.107 | 101.0 (99.9) | 50 | 17 (15) | 18 (6) | Buformin |
| Nicotine | 3.6 | 163.01 | 130.0 (117.0) | 56 | 27 (35) | 8 (15) | Buformin |
| Propofol | 4.1 | 179.179 | 101.0 (79.0) | 36 | 9 (11) | 6 | Buformin |
| Thiamine | 2.8 | 265.105 | 122.1  (143.8) | 50 | 25  (19) | 10 | Buformin |
| Tyramine | 4.1 | 138.1 | 121.1 | 43 | 14 | 12 | Buformin |
| 8% organic additive (91.9% ddH_2_O, 0.1% formic acid, 6.9% acetonitrile, 1.1% methanol) | | | | | | | |
| Acetyl-L-carnitine | 3.0 | 204.1 | 85.0 (134.93) | 60 | 27 | 6 | Ranitidine-d6 |
| Acyclovir | 3.9 | 225.98 | 151.9 (134.93) | 46 | 17 (40) | 10 (8) | Ranitidine-d6 |
| Ephedrine | 4.2 | 166.02 | 148.1 (133.0) | 41 | 17 (27) | 9 (8) | Ranitidine-d6 |
| Hydrocodone | 6.8 | 300.472 | 283.1 (133.1) | 56 | 15 (21) | 26 (28) | Ranitidine-d6 |
| Hydromorphone | 4.2 | 286.2 | 185.2 (157.1) | 110 | 43 (55) | 5 (10) | Ranitidine-d6 |
| Morphine | 3.8 | 166.02 | 148.1 (133.0) | 41 | 17 (27) | 9 (8) | Ranitidine-d6 |
| Ranitidine-d6 | 4.4 | 321.2 | 176.0 (130.1) | 65 | 25 (35) | 15 |  |
| Sumatriptan | 5.0 | 296.2 | 58.2 (251.2) | 50 | 30 (24) | 12 | Ranitidine-d6 |
| TEA (Tetraethylammonium) | 3.8 | 130.247 | 86.1 (58.2) | 61 | 30 (45) | 16 (10) | Ranitidine-d6 |
| 20% organic additive (79.9% ddH_2_O, 0.1% formic acid, 17.2% acetonitrile, 2.8% methanol) | | | | | | | |
| Amisulpride | 3.8 | 370.2 | 242.1 (195.8) | 90 | 38 (52) | 15 (15) | Fenoterol-d6 |
| Amitriptyline-d6 | 4.0 | 284.3 | 91.039 | 20 | 30 | 15 |  |
| Amphetamine | 3.6 | 136.0 | 91.0 (119.0) | 41 | 21 (13) | 16 (14) | Fenoterol-d6 |
| Articaine | 4.2 | 285.203 | 86.2 (253.1) | 63 | 21 (17) | 16 (24) | Fenoterol-d6 |
| Atropine | 3.7 | 290.2 | 142.2 (124.2) | 100 | 45 (33) | 12 | Fenoterol-d6 |
| Clonidine | 3.6 | 230.0 | 44.0 (213.0) | 85 | 46 (34) | 15 | Fenoterol-d6 |
| Clozapine | 7.5 | 327.154 | 270.1 (192.2) | 101 | 31 (57) | 18 (12) | Fenoterol-d6 |
| Dobutamine | 3.2 | 302.202 | 137.0 (106.9) | 66 | 30 (37) | 10 (6) | Fenoterol-d6 |
| Emtricitabine | 3.7 | 247.95 | 129.97 | 44 | 15 | 8 | Fenoterol-d6 |
| Fenoterol-d6 | 3.6 | 310.3 | 109.1 (141.0) | 70 | 40 (26) | 12 |  |
| Galantamine | 2.3 | 288.221 | 213.1 (231.2) | 86 | 31 (23) | 14 (16) | Fenoterol-d6 |
| MDMA (3,4-Methylen-dioxy-N-methylamphetamine) | 3.6 | 193.9 | 163.0 (104.9) | 41 | 17 (33) | 10 (6) | Fenoterol-d6 |
| MDAI (5,6-Methylenedioxy-2- aminoindane) | 3.5 | 177.97 | 161.0 (131.0) | 43 | 17 (27) | 10 (16) | Fenoterol-d6 |
| Medetomidine | 5.7 | 201.243 | 95.1 (68.2) | 71 | 23 (49) | 18 (12) | Fenoterol-d6 |
| Meperidine | 5.3 | 248.394 | 174.1 (70.1) | 51 | 29 (47) | 12 | Fenoterol-d6 |
| Methylphenidate | 4.4 | 234.174 | 84.1 (91.1) | 71 | 27 (73) | 6 (8) | Fenoterol-d6 |
| Metoprolol | 4.6 | 268.2 | 116.1 (74.0) | 86 | 27 (35) | 8 (14) | Fenoterol-d6 |
| MPP+ (1-Methyl-4-phenylpyridinium) | 2.7 | 170.016 | 128.1 (154.0) | 100 | 42 (43) | 8 (10) | Fenoterol-d6 |
| Nalmefen | 4.2 | 340.252 | 322.2 (55.1) | 61 | 29 (63) | 10 | Fenoterol-d6 |
| Naloxone | 3.5 | 328.2 | 310.3 (253.2) | 86 | 27 (35) | 20 (16) | Fenoterol-d6 |
| Nimodipine | 3.8 | 419.2 | 402.3 (45.2) | 75 | 17 (84) | 12 (8) | Fenoterol-d6 |
| Nortilidine | 3.9 | 260.3 | 155.1 (115.1) | 61 | 25 (67) | 12 (10) | Fenoterol-d6 |
| Oxycodone | 3.6 | 316.3 | 298.2 (256.2) | 91 | 27 (35) | 10 (8) | Fenoterol-d6 |
| Rasagiline | 3.5 | 172.109 | 117.0 (56.1) | 40 | 15 (11) | 8 (10) | Fenoterol-d6 |
| Scopolamine | 2.9 | 304.3 | 138.1 | 80 | 30 | 15 | Fenoterol-d6 |
| Tranylcypromine | 3.6 | 134.048 | 117.0 (115.0) | 41 | 13 (27) | 8 | Fenoterol-d6 |
| Tryptophan-d5 | 3.7 | 210.144 | 192.1 (193.1) | 39 | 14 (9) | 12 | Fenoterol-d6 |
| Varenicline | 3.6 | 212.2 | 169.1 (183.1) | 95 | 29 (31) | 12 | Fenoterol-d6 |
| 35% organic additive (64.9% ddH_2_O, 0.1% formic acid, 30% acetonitrile, 5% methanol) | | | | | | | |
| Biperiden | 4.3 | 312.237 | 98.2 (70.0) | 80 | 36 (72) | 18 (12) | Bupivacaine |
| Bupivacaine | 3.5 | 289.248 | 140.1 (84.2) | 75 | 30 (58) | 8 (16) | Amitriptylin-d6 |
| Buspirone | 3.4 | 386.212 | 122.2 (95.1) | 100 | 42 (71) | 10 (8) | Bupivacaine |
| Cocaine | 3.4 | 304.338 | 182.0 (77.0) | 41 | 27 (77) | 12 (14) | Bupivacaine |
| Daunorubicine | 4.2 | 528.2 | 321.0 (363.0) | 63 | 25 (19) | 10 (11) | Bupivacaine |
| Diphenhydramine | 3.6 | 256.2 | 167.0 (152.0) | 46 | 17 (49) | 10 | Bupivacaine |
| Fentanyl | 3.5 | 337.35 | 188.2 (76.9) | 91 | 33 (107) | 12 (6) | Bupivacaine |
| Fluoxetine | 4.2 | 310.159 | 44.1 (148.1) | 56 | 39 (13) | 8 (10) | Bupivacaine |
| Ketamine | 2.6 | 239.026 | 126.1 (125.1) | 50 | 37 (34) | 8 | Bupivacaine |
| Lidocaine | 3.3 | 235.1 | 86.2 (158.9) | 71 | 26 (11) | 16 (12) | Bupivacaine |
| Mepivacaine | 3.3 | 247.2 | 98.0 | 77 | 28 | 5 | Bupivacaine |
| Pyrilamine | 2.5 | 286.236 | 121.2 (241.2) | 56 | 33 (19) | 22 (22) | Bupivacaine |
| Rivastigmine | 3.3 | 251.1 | 206.1 (86.1) | 51 | 19 (33) | 14 (16) | Bupivacaine |
| Ropivacaine | 3.4 | 275.267 | 126.2 (84.1) | 81 | 29 (59) | 8 (16) | Bupivacaine |
| Sufentanil | 3.4 | 387.152 | 238.1 (110.9) | 81 | 27 (53) | 8 | Bupivacaine |
| Tetracaine | 3.0 | 265.1 | 176.1 (72.3) | 61 | 20 (37) | 10 (14) | Bupivacaine |
| Venlafaxine | 3.35 | 278.2 | 58.1 (121.0) | 65 | 47 (39) | 10 (15) | Bupivacaine |
| 50% organic additive (49.9% ddH_2_O, 0.1% formic acid, 42.9% acetonitrile, 7.1% methanol) | | | | | | | |
| Estrone-3-sulfate | 8.6 | 348.917 | 269.3 (145.1) | -100 | -45 (-74) | -15 (-9) | Ibuprofen |
| Fexofenadine | 3.1 | 502.322 | 466.4 (171.1) | 100 | 37 (53) | 14 (10) | Tamoxifen |
| Ibuprofen | 5.8 | 204.9 | 161.1 (159.1) | -50 | -10 | -9 |  |
| Tamoxifen | 4.6 | 372.232 | 71.9 (70.0) | 90 | 47 (75) | 14 (6) |  |

*Abbreviations: RT, retention time; Q1/Q3 mass, first/third quadrupole mass filter with quantifier and (qualifier); DP, declustering potential; CE, collision energy; CXP, collision cell exit potential.

API 4000 tandem mass spectrometer (AB SCIEX, Darmstadt, Germany).
