# Supplementary figures and images for: Several orphan solute carriers functionally identified as organic cation transporters: Substrates specificity compared with known cation transporters
Source: J Biol Chem. 2024 Aug 3;300(9):107629. doi: 10.1016/j.jbc.2024.107629 (PMC11406361; doi:10.1016/j.jbc.2024.107629)

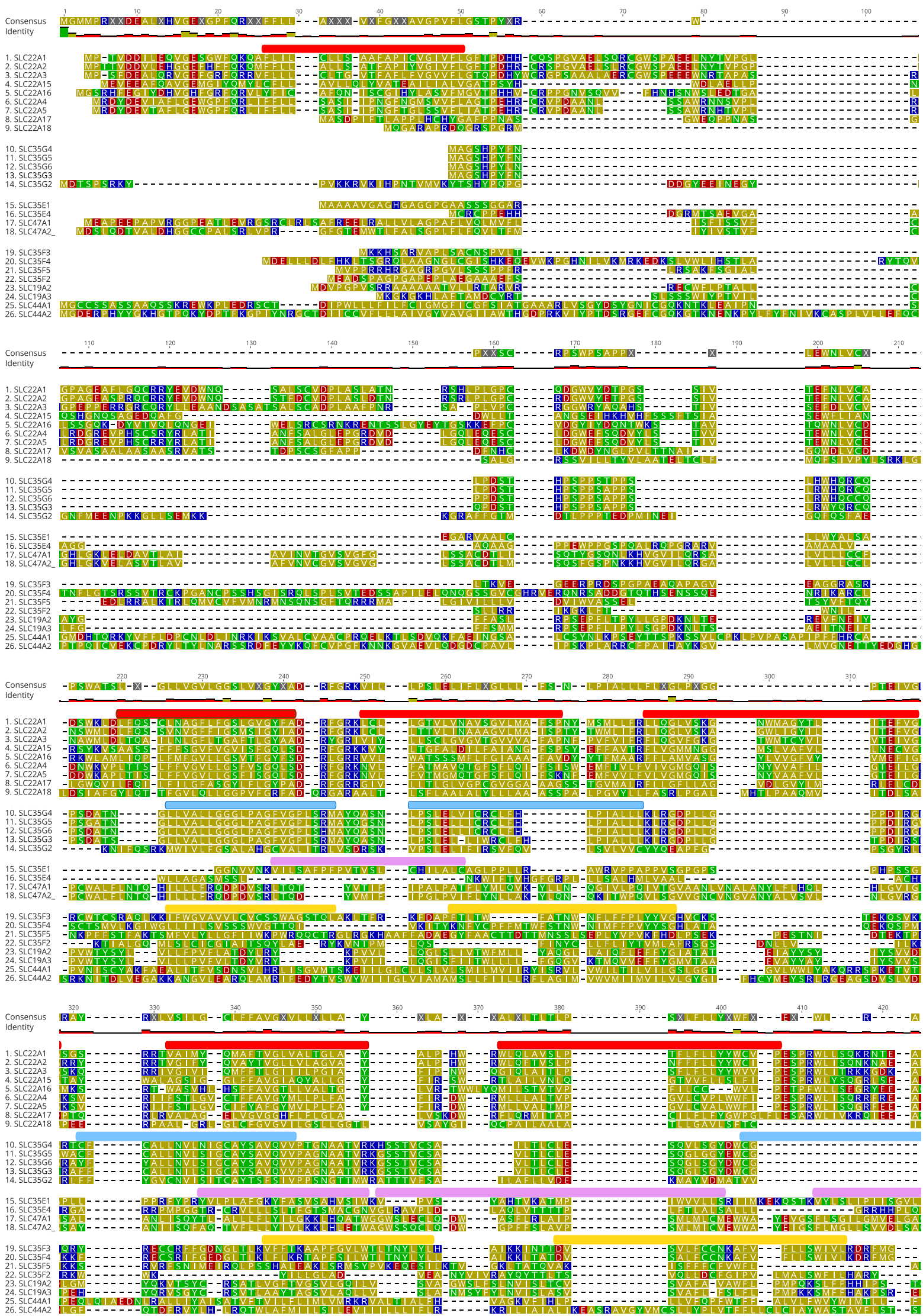

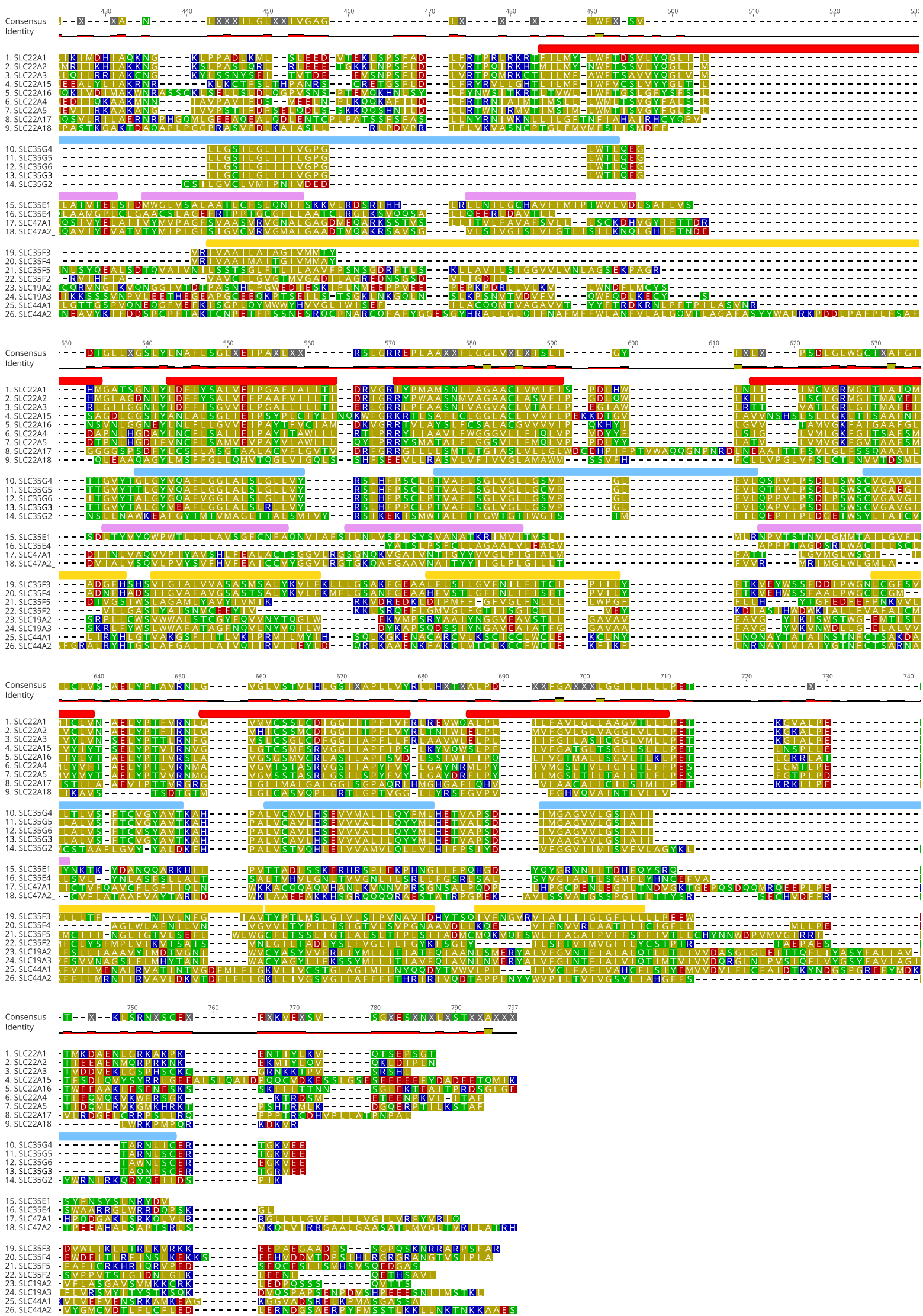

Supplement: Supplement Figure S1 [file mmc2.pdf]

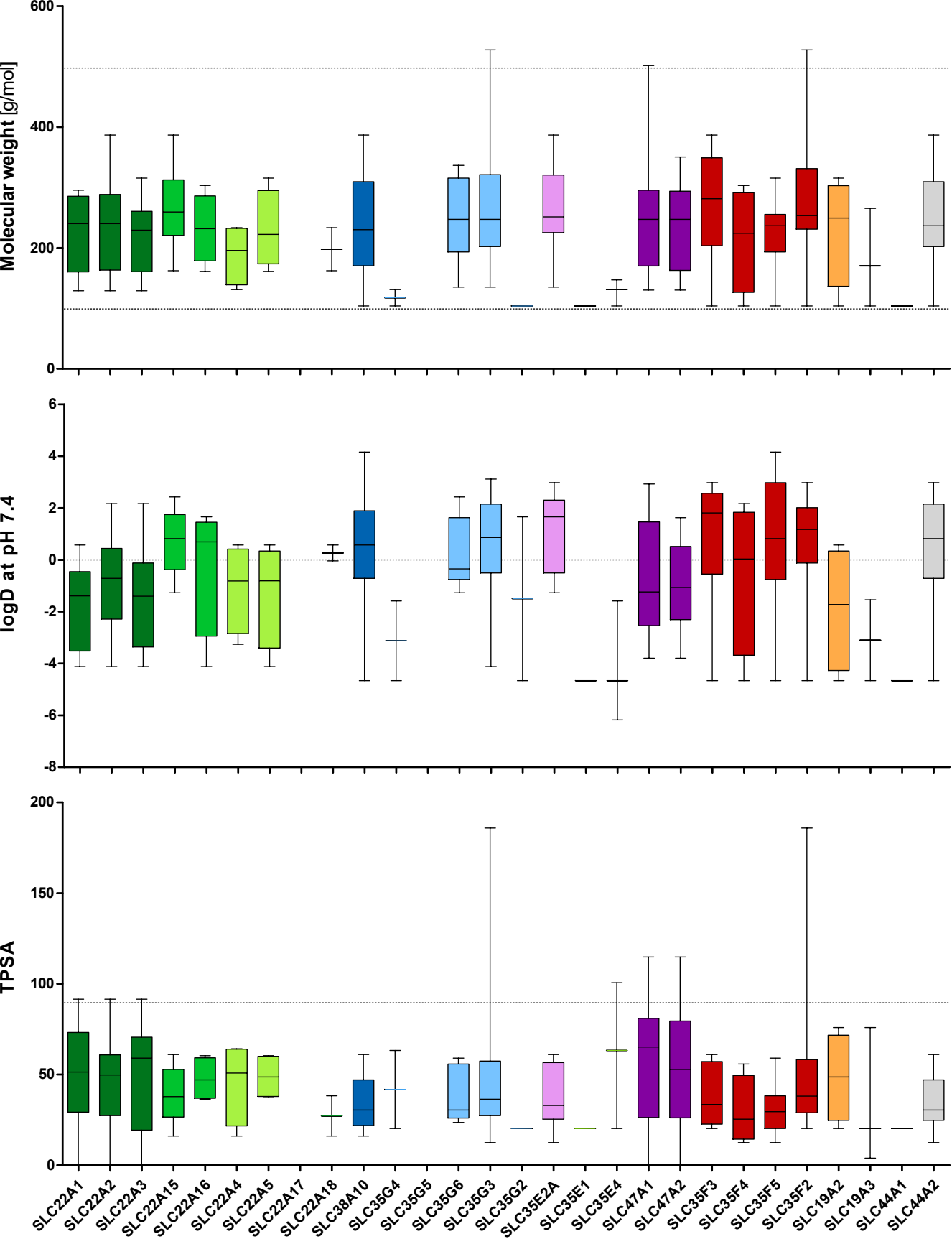

Supplement: Supplement Figure S2 [file mmc3.pdf]

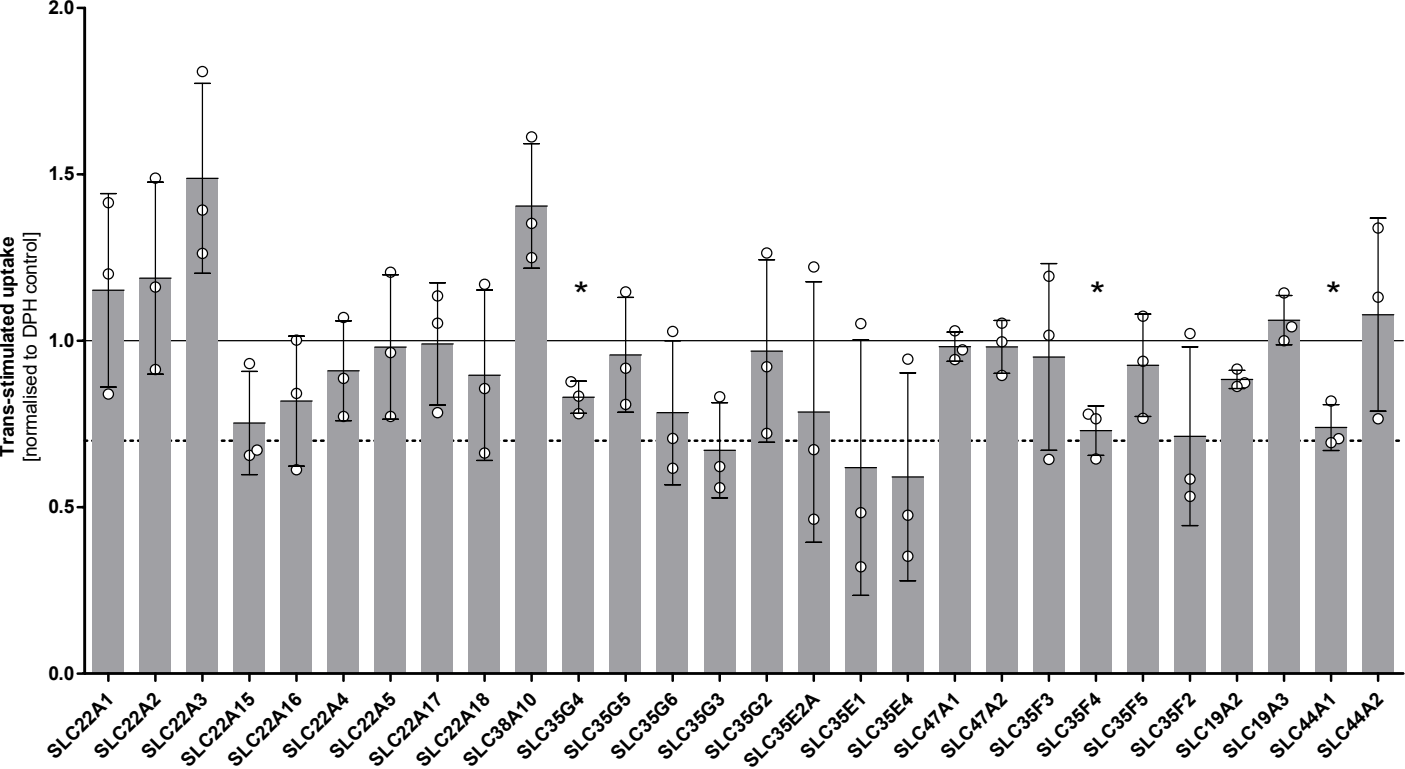

Supplement: Supplement Figure S3 [file mmc4.pdf]

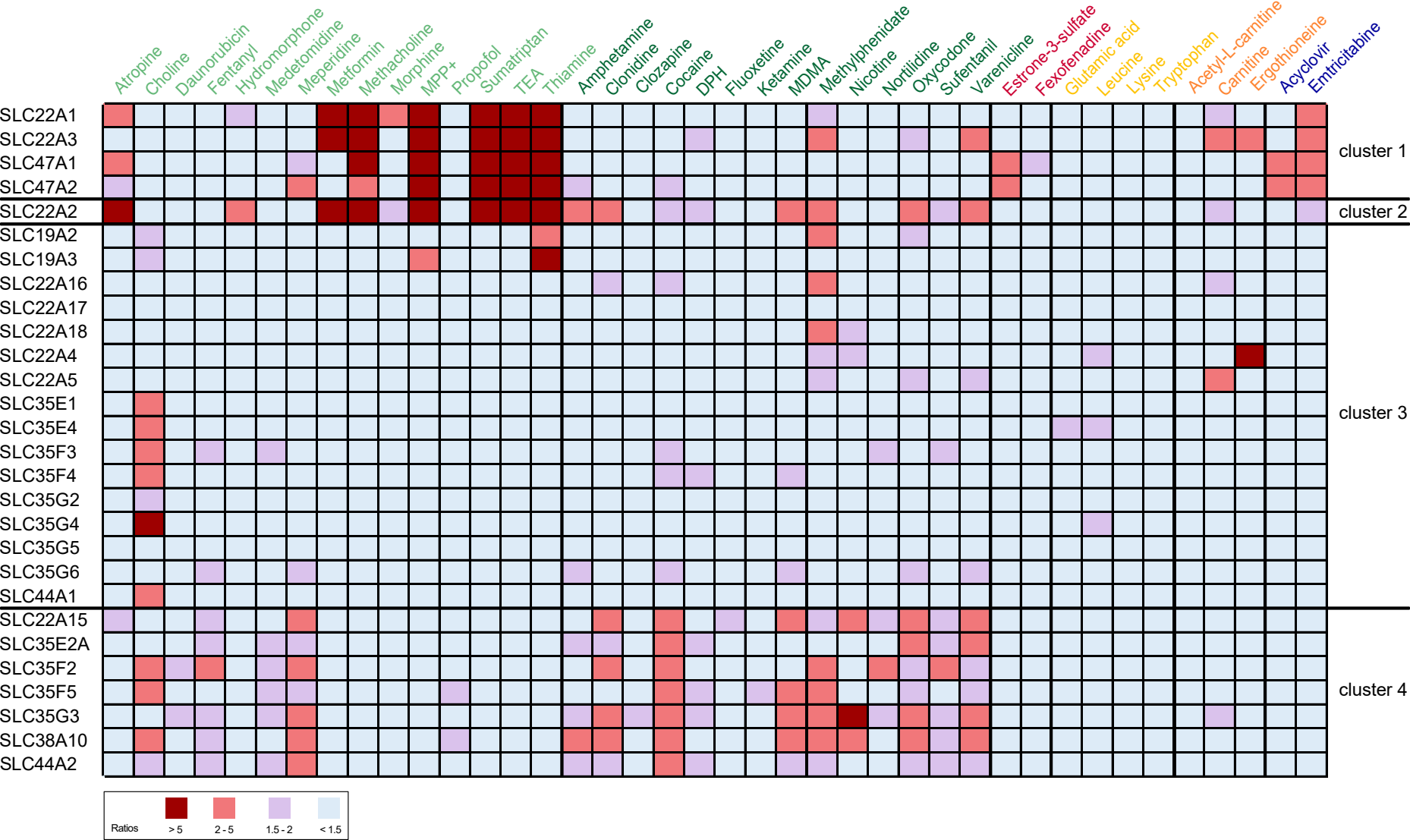

Supplement: Supplement Figure S4 [file mmc5.pdf]
