# Supplementary material for: Several orphan solute carriers functionally identified as organic cation transporters: Substrates specificity compared with known cation transporters
Source: J Biol Chem. 2024 Aug 3;300(9):107629. doi: 10.1016/j.jbc.2024.107629 (PMC11406361; doi:10.1016/j.jbc.2024.107629)
Supplement: Supplement Figure S6 [file mmc7.pdf]

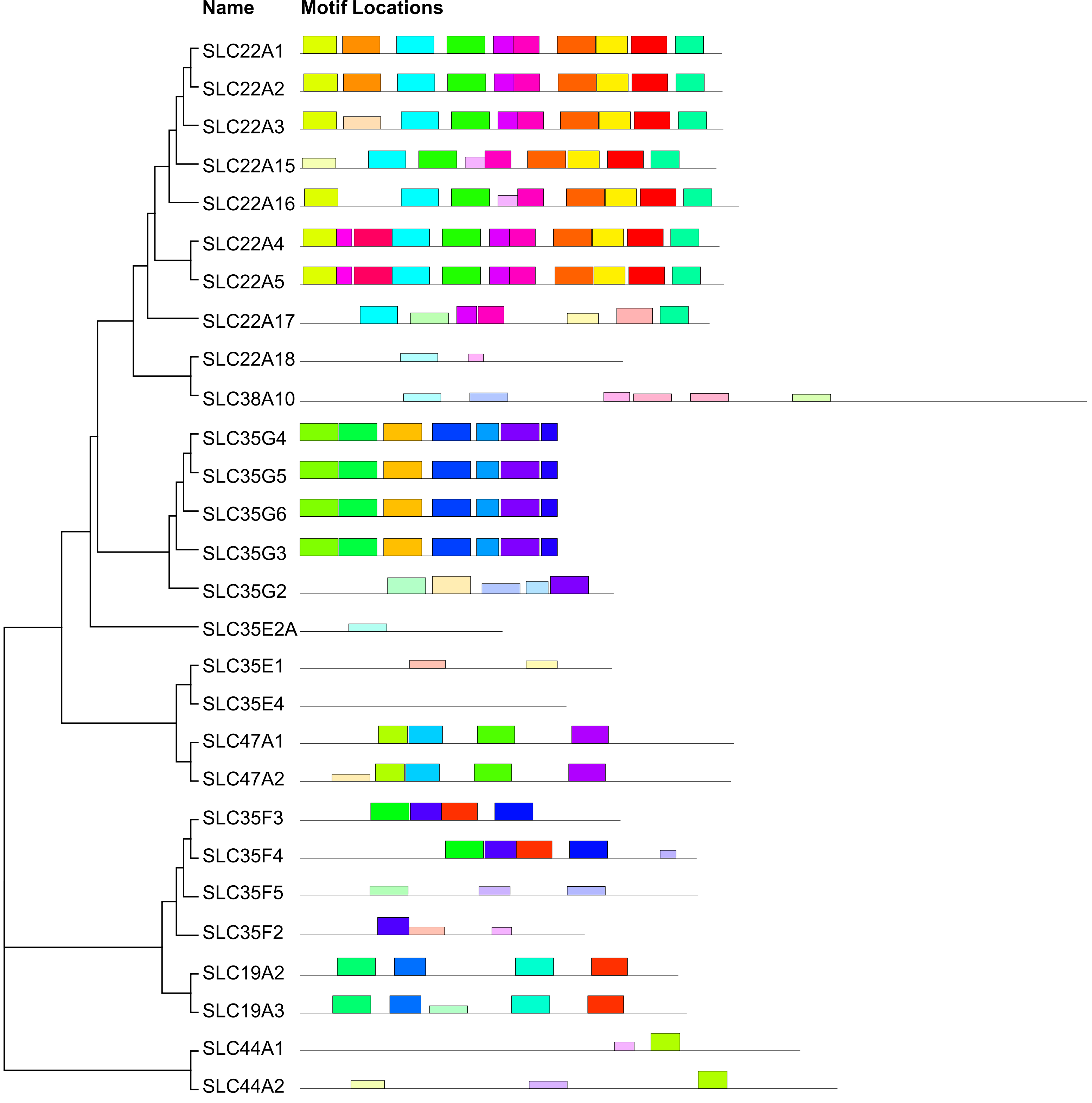

Motif	Symbol	Motif Consensus
1.		MLGKFGITAAFEIVYLYTAELYPTVVRNLGVGVCSTLCRIGGIIAPF
2.		DTYLSTIVTEWNLVCEDSWKVDLTQSLFFVGVLLGSFTLGYLADRFGRK
3.		MAGSHPYFNLPDSTHPSPPSAPPSLRWHQRCQPSDATNGLLVALLGGGLP
4.		VSFTCVGYAVTKAHPALVCAVLHSEVVVALILQYYMLHETVAPSDIVGAG
5.		NVLSIGCAYSAVQVVPAGNAATVRKGSSTVCSAVLTLCLESQGLSGYDWC
6.		GFVGPLSRMAYQASNLPSLELLICRCLFHLPIALLLKLRGDPLLGPPDIR
7.		PGLWTLQEGTTGVYTALGYVQAFLGGLALSLGLLVYRSLHFPSCLPTVAF
8.		FIPESPRWLISQGRFEEAZKIJRKIAKKNGKLVP
9.		RTPQIRKHTLILMYJWFTSSVGYQGLSLHAGNLGGNIYLBFFLSALVEIP
10.		DEVLEHVGEFGPFQRLIFFLLALSSIPFGFJYVGIVFLGFTPDH
11.		AFSPNYEMFVVFRFLVGMVSKGNWVVAFVLITEFVGKKYRRIVGILGQMF
12.		LPYIVFGALALLAGGLTLLLPETKGKALPETJEDAEK
13.		GSVPGLFVLQSPVLPSDLLSWSCVGAVGI
14.		GSIAIITARNLSCERTGKVEE
15.		AYFIPDWRWLQLAITLPGFLFLLYYW
16.		RDGREVPHSCRRYRLATIANFSALGLEPGRDVDLGQLEQESCLDGWEFSQ
17.		NKAWVFLLSWIVFKDRYMGLRTIAAFQAAAGJVMERYADVFGVNSFI
18.		ELSQRCGWSPAEELNYTVPGPGPAGEAFPGQCRRYEVDWNQSAFDCVDP
19.		YFJALJTJDRLGRRYPLAASLFLAGAACLVVMFIPEDLYWL
20.		YGSQNLKHVGNILQRCALVLLLCCFPCWEKFLNTQNIL
21.		YILGKKLHQETWGGWSLZCLQDWGPFFRLAIPSMLMJCMEWWAYEIGSF
22.		FDCPFFLTWFATNWNFLFFPLYYVGHLCKAQEKQSPKKKFRECCRFFGED
23.		EPFLIPYLLGPDKNLTEREIFNEIFPVWTYSYLVLLFPVFLATDYLRYKP
24.		NDFKECYSSKRLFCWSLWWAFATCGFNQVLNYVQGLWEKKAPSRDAAIYN
25.		FRQDPDVSRLTQDYVMIFIPGLPVIFLYNLQVKYLQNQGIVWPQ
26.		QFLEFFYGIVTAAEIAYYAYIYSVVDPEHYQKVSGYCRSVT
27.		GSAKFGEAAHFLSILGFFNJJFISCIPIILYFTKVEHWSSFDDJPWGCLC
28.		TLKLFWWKAAPFGILWTLTNYLYLRAJKKJTATDVSVLFCC
29.		KDQLGHIFTNDEDIINLVAQVLPIYAVFHLFEAJCCVYGGVLRGSGKQ
30.		RCRVPDAANLSSAWRNNSVP
