# Supplementary material for: Several orphan solute carriers functionally identified as organic cation transporters: Substrates specificity compared with known cation transporters
Source: J Biol Chem. 2024 Aug 3;300(9):107629. doi: 10.1016/j.jbc.2024.107629 (PMC11406361; doi:10.1016/j.jbc.2024.107629)
Supplement: Supplement Figure S5 [file mmc6.pdf]

	SLC22A1	SLC22A2	SLC22A3	SLC22A15	SLC22A16	SLC22A4	SLC22A5	SLC22A17	SLC22A18	SLC38A10	SLC35G4	SLC35G5	SLC35G6	SLC35G3	SLC35G2	SLC35E2A	SLC35E1	SLC35E4	SLC47A1	SLC47A2	SLC35F3	SLC35F4	SLC35F5	SLC35F2	SLC19A2	SLC19A3	SLC44A1	SLC44A2
SLC22A1		0.78	0.61	-0.21	-0.01	-0.04	-0.20	0.05	-0.33	-0.27	0.01	0.21	-0.06	0.08	0.18	-0.28	-0.23	-0.24	0.43	0.50	-0.36	-0.14	-0.23	-0.07	0.00	0.24	-0.12	-0.20
SLC22A2			0.50	0.15	0.09	0.06	-0.01	-0.21	-0.29	0.02	-0.16	0.37	0.22	0.39	-0.01	0.01	-0.16	-0.32	0.35	0.61	-0.12	-0.15	-0.04	0.16	0.12	0.22	-0.04	0.06
SLC22A3				-0.26	-0.08	0.24	0.14	0.15	-0.20	-0.44	0.37	0.14	-0.19	-0.11	0.49	-0.35	-0.07	0.13	0.31	0.38	-0.21	-0.13	-0.18	-0.19	-0.04	0.38	-0.08	-0.29
SLC22A15					0.60	0.43	0.38	-0.26	0.40	0.77	-0.28	0.39	0.68	0.77	-0.02	0.75	0.33	0.18	-0.02	0.05	0.59	0.41	0.57	0.79	0.42	0.11	0.48	0.74
SLC22A16						0.34	0.28	0.21	0.56	0.60	-0.30	0.61	0.74	0.69	0.03	0.55	0.18	0.10	0.02	-0.01	0.41	0.23	0.21	0.52	0.43	0.22	0.59	0.61
SLC22A4							0.62	-0.02	0.34	0.40	0.07	0.35	0.39	0.36	0.30	0.29	0.25	0.24	0.02	-0.05	0.20	0.21	0.41	0.35	0.26	0.12	0.26	0.42
SLC22A5								-0.05	0.34	0.37	0.05	0.46	0.48	0.39	0.18	0.47	0.33	0.37	-0.08	0.05	0.48	0.34	0.43	0.42	0.33	0.28	0.31	0.41
SLC22A17									0.28	-0.20	0.49	0.14	-0.12	-0.18	0.41	-0.21	0.06	0.31	0.18	0.08	0.05	0.00	-0.25	-0.03	0.12	0.43	0.27	-0.12
SLC22A18										0.42	0.11	0.14	0.21	0.22	0.17	0.31	0.12	0.13	-0.18	-0.43	0.25	0.20	0.25	0.32	0.40	0.24	0.29	0.45
SLC38A10											-0.43	0.43	0.77	0.70	-0.12	0.79	0.44	0.04	-0.17	-0.09	0.53	0.47	0.69	0.67	0.62	0.14	0.55	0.89
SLC35G4												-0.29	-0.54	-0.42	0.78	-0.45	-0.06	0.49	0.12	-0.05	0.04	0.12	-0.08	-0.14	-0.12	0.35	0.00	-0.27
SLC35G5													0.72	0.74	-0.03	0.54	0.25	0.05	0.21	0.35	0.23	0.01	0.16	0.40	0.40	0.37	0.41	0.55
SLC35G6														0.81	-0.25	0.73	0.32	0.08	-0.02	0.18	0.57	0.31	0.39	0.67	0.42	0.12	0.62	0.72
SLC35G3															-0.15	0.71	0.18	-0.03	0.06	0.20	0.43	0.25	0.43	0.63	0.35	0.16	0.48	0.72
SLC35G2																-0.14	0.25	0.56	0.23	0.05	0.10	0.29	0.13	0.07	0.12	0.43	0.20	0.01
SLC35E2A																	0.58	0.24	-0.11	0.04	0.59	0.42	0.58	0.73	0.51	0.20	0.39	0.75
SLC35E1																		0.52	-0.03	-0.01	0.45	0.41	0.36	0.43	0.51	0.38	0.38	0.38
SLC35E4																			0.15	0.00	0.54	0.41	0.15	0.41	0.11	0.43	0.45	0.14
SLC47A1																				0.65	-0.09	-0.32	-0.23	0.06	0.00	0.27	0.12	-0.17
SLC47A2																					0.05	-0.16	-0.14	0.18	0.02	0.25	0.06	-0.06
SLC35F3																						0.59	0.46	0.79	0.33	0.31	0.65	0.50
SLC35F4																							0.69	0.50	0.33	0.17	0.42	0.51
SLC35F5																								0.50	0.47	0.16	0.31	0.71
SLC35F2																									0.44	0.34	0.62	0.62
SLC19A2																										0.60	0.42	0.60
SLC19A3																											0.46	0.24
SLC44A1																												0.55
SLC44A2																												

Substrate correlation: -1.0 · 0.0 · 1.0
